# Histotaphonomic analysis of bone bioerosion reveals a regional framework of diverse deathways in the Neolithic of Southeast Italy

**DOI:** 10.1371/journal.pone.0304058

**Published:** 2024-06-06

**Authors:** Jess E. Thompson, Sofia Panella, Thomas J. Booth, Silvia Soncin, Tonko Rajkovaca, Maria Giovanna Belcastro, Eugenia Isetti, Valentina Mariotti, Italo Maria Muntoni, Francesca Radina, Sandra Sivilli, Antonella Traverso, Mary Anne Tafuri, John E. Robb

**Affiliations:** 1 McDonald Institute for Archaeological Research, Cambridge, United Kingdom; 2 Darwin College, Cambridge, United Kingdom; 3 Department of Environmental Biology and Mediterranean bioArchaeological Research Advances (MAReA) Centre, Sapienza University of Rome, Rome, Italy; 4 Francis Crick Institute, London, United Kingdom; 5 Department of Archaeology, University of Cambridge, Cambridge, United Kingdom; 6 Department of Biological, Geological and Environmental Sciences, University of Bologna, Bologna, Italy; 7 Istituto Italiano per l’Archeologia Sperimentale, Genova, Italy; 8 Soprintendenza Archeologia, Belle Arti e Paesaggio per le Province di Barletta-Andria-Trani e Foggia, Italy; 9 Soprintendenza Archeologia, Belle Arti e Paesaggio per la Città Metropolitana di Bari, Bari, Italy; 10 Independent Researcher, Bari, Italy; 11 Direzione Regionale, Musei Liguria, Genova, Italy; University of Otago, NEW ZEALAND

## Abstract

The wide diversity of Neolithic funerary practices is increasingly recognised. In Southeast Italy, recent studies have drawn attention to the co-existence of multiple ways of treating the dead within single sites and across the region. In this study, we address how such diverse deathways form a regional framework of ritual practice through histotaphonomic analysis of bone bioerosion. Samples were obtained from articulated, semi-articulated and disarticulated remains from four sites in Apulia which each presented different modes of treatment and disposal of the dead. Bone thin sections were analysed by light microscopy to characterise microstructural preservation through features including bacterial bioerosion, staining, inclusions, and Wedl tunnelling. We investigate the early post-mortem histories of individuals whose remains ended up in various states of dis/articulation and diverse depositional contexts. Disarticulated remains frequently displayed arrested or extensive bacterial bioerosion, which was also found in articulated and semi-articulated skeletons. Additionally, remains deposited in similar contexts, as well as articulated and disarticulated remains deposited together in the same context, often showed different histotaphonomic characteristics, suggesting diverse early post-mortem trajectories. As a result, we argue that Neolithic deathways in southeastern Italy incorporated a high level of diversity in the early post-mortem treatment of the body. A framework for funerary practices emerges, whereby disarticulated remains probably originated from bodies which had been buried previously and subjected to varying extents of shelter, exposure to invertebrates, and duration of burial. However, we acknowledge the ongoing research into the origins of bacterial bioerosion and the problem of equifinality, which leaves open the possibility for further scenarios of early post-mortem treatment.

## 1. Introduction

The Neolithic in peninsular Italy appeared earliest in the Apulian Tavoliere and the Salento Peninsula, just before the turn of the 6^th^ millennium BC, probably following small-scale migrations of groups across the Adriatic Sea [1–4]. The southeast of the Italian Peninsula was rapidly densely inhabited by communities who constructed large villages inland and along the coast [5, 6]. These villages became a focal point for burying and disposing of the dead, both during their main phase of occupation and after their abandonment [1, 7]. Outside of these, sporadic occupation, ritual activities, and funerary rites also occurred in caves [8]. Funerary treatment—the modes of deposition of bodies and body parts, and the features in which they were placed—varied widely across these sites. Simple, undisturbed burials of flexed skeletons (usually lacking grave goods or with a single pot), are attested alongside disturbed and incomplete burials from which bones have been removed, double or multiple burials in settlement features or cists, groups of re-deposited bones, scattered and disarticulated bones in village ditches or occupation levels, burials or disarticulated bones displaying cutmarks, and occasional cremated bone fragments [1, 9–11]. The variety of funerary practices evident across an otherwise quite culturally homogeneous region raises questions regarding the social, political and ritual framework guiding deathways. At a micro-scale, what were the post-mortem histories of bones which were circulated before secondary burial? How did diverse deathways within the same archaeological features relate to one another? And, more broadly, how did funerary practices connect across communities in southeast Italy?

Histotaphonomic analysis of bone microstructural preservation is a useful tool for investigating bone diagenesis and early postmortem histories [12, 13]. Several main diagenetic pathways are responsible for the degradation of bone: bacterial and fungal attack, the biological destruction of bone’s organic and mineral phases; accelerated collagen hydrolysis, the chemical deterioration of the organic phase; and mineral dissolution, the chemical deterioration of the organic and mineral phases [14–17]. As these processes alter bone’s microstructural properties, they can be investigated at a histological level by assessing the presence of micro-foci of destruction (MFD) and the preservation of bone collagen, alongside other taphonomic factors. MFD are classified according to their morphological appearance [18]. Wedl MFD are narrow branching tunnels which pervade through the cortex. They have been variably attributed to cyanobacteria in aquatic environments and saprophytic fungi in open terrestrial contexts, although there is some debate over the latter and whether fungi are capable of bioeroding bone [12, 19–22]. Non-Wedl MFD describes defects of diverse forms which erode mineral within and around osteons, and are attributed to bacteria [18, 23, 24]. Bacterial bioerosion is the most common mode of diagenesis affecting archaeological human bone [14, 15, 25, 26].

Two models have been proposed for the origin of the microbes responsible for bacterial bioerosion. The endogenous model attributes the destruction of bone to the transmigration of gut bacteria during the putrefactive stage of decomposition [14, 15, 27–30]. Conversely, the exogenous model identifies microbes in the soil surrounding the body as the primary agents of bioerosion [20, 31–35]. Although, a symbiotic relationship between the two may also be considered [22, 36, 37]. Some have suggested that the location of bioerosion within bone may reflect its origins, with focal destruction on the periosteal band more likely caused by soil micro-organisms, and endosteal destruction by endogenous bacteria [20, 32, 35]. However, this does not appear to be supported by some experimental studies using pig skeletal remains [38].

An endogenous model of bacterial bioerosion has been invoked previously to explain the results of multiple large-scale surveys of archaeological bone which found a strong correlation between bacterial bioerosion and early taphonomy (e.g. butchery, pre-depositional disarticulation, intact burial), but little correlation with burial conditions outside of anoxic or waterlogged environments [14, 39–41]. However, actualistic real-time experiments investigating this association have produced contradictory results regarding an endogenous model of bioerosion [29, 35, 38, 42]. Recent histotaphonomic research on five donated human bodies deposited in different conditions found no onset of bacterial bioerosion after 30 months; in this environment (Texas, USA), decomposition was not immediately associated with the migration of bacteria into bone [36]. The authors suggest that the relationship between decomposition and the burial microbiome could be an important influencing factor, while further consideration of temperature, climate, soil pH, and medical histories, are needed for fuller interpretation of histotaphonomic data. Histological analysis of bones from pig carcasses and a human body which had been exposed, versus defleshed and buried cow bones, showed bacterial bioerosion was present only in the cow bones which had been surrounded by soil [35]. Yet, differential intensity of bacterial bioerosion in bones deposited in the same soils or archaeological features, as well as an association with other markers of early taphonomy, is still frequently evidenced in archaeological studies [41, 43, 44]. These studies suggest that some bone bioerosion may be caused by bacteria in soil but is still influenced by a range of factors beyond the burial environment. This tension between results from small-scale actualistic experiments and large-scale surveys of archaeological bone lies at the heart of ongoing debates around the aetiology of bacterial bone bioerosion. The key may be in exploring scenarios which could explain both sets of observations. For instance, if soil bacteria are more attracted to or are more able to exploit bone that is surrounded by soft tissue [45].

Most research into the origins of non-Wedl MFD has suggested their presence in bone is related to the process of decomposition [14, 29, 40]. When bacterial bioerosion is absent from articulated skeletons recovered from well-drained, aerobic contexts, it is often inferred that gut bacteria had not yet developed, for example as a result of age (i.e. foetal or stillborn babies) or because decomposition was affected by exceptional methods of soft tissue preservation such as mummification [46–50]. As decomposition and diagenetic changes are generally thought to be incurred early in the post-mortem interval, bacterial bioerosion may provide finer-grained taphonomic insights into funerary treatment [30, 51–53]. Therefore, analysis of bacterial bioerosion has been suggested to be informative for broadly distinguishing between funerary treatments which have exposed the dead body to different extents of putrefaction or bacterial bodily decomposition, including decomposition by soil micro-organisms [14, 28, 30, 40, 46, 54]. Given the contradictory evidence from recent experimental research, which suggests that bacterial bioerosion and putrefaction are not linked [35, 36], further interpretations may be worth considering. For example, it is possible that perfect histological preservation instead indicates exposure or burial in a more sterile environment for a short period post-mortem. Primary burial, in which the dead body progresses through full decomposition in a grave or other feature surrounded by soil, is expected to result in extensive bacterial bioerosion, and this is commonly observed in bones from articulated skeletons that were probably buried whole and intact soon after death [14, 39, 40]. Diverse post-mortem treatments such as coffin burial, excarnation, defleshing, and disarticulation may lead to absent or partial bacterial bioerosion, related to the ecology of the depositional environment, perhaps also in tandem with the location of the body during the decomposition process [55–57].

Prehistoric funerary practices have recently been the focus of a large number of histotaphonomic studies, from contexts as wide-ranging as disturbed primary burials and secondary depositions at Neolithic Çatalhöyük [44, 57], Neolithic megalithic burials in southwest Sweden [58], Neolithic primary burials and Mycenaean and Minoan collective tombs in the Aegean [43, 56], and burials and disarticulated bones from Bronze and Iron Age contexts in Britain [47, 54, 59]. Until now, histotaphonomic analysis has not been applied to prehistoric skeletal remains from Italy. This study aims to investigate early post-mortem funerary treatments through histotaphonomic preservation across a range of contexts and, in so doing, to better understand the relationship between different modes of burying and disposing of the dead body during the Neolithic in Southeast Italy.

## 2. Archaeological context

**Fig 1:**
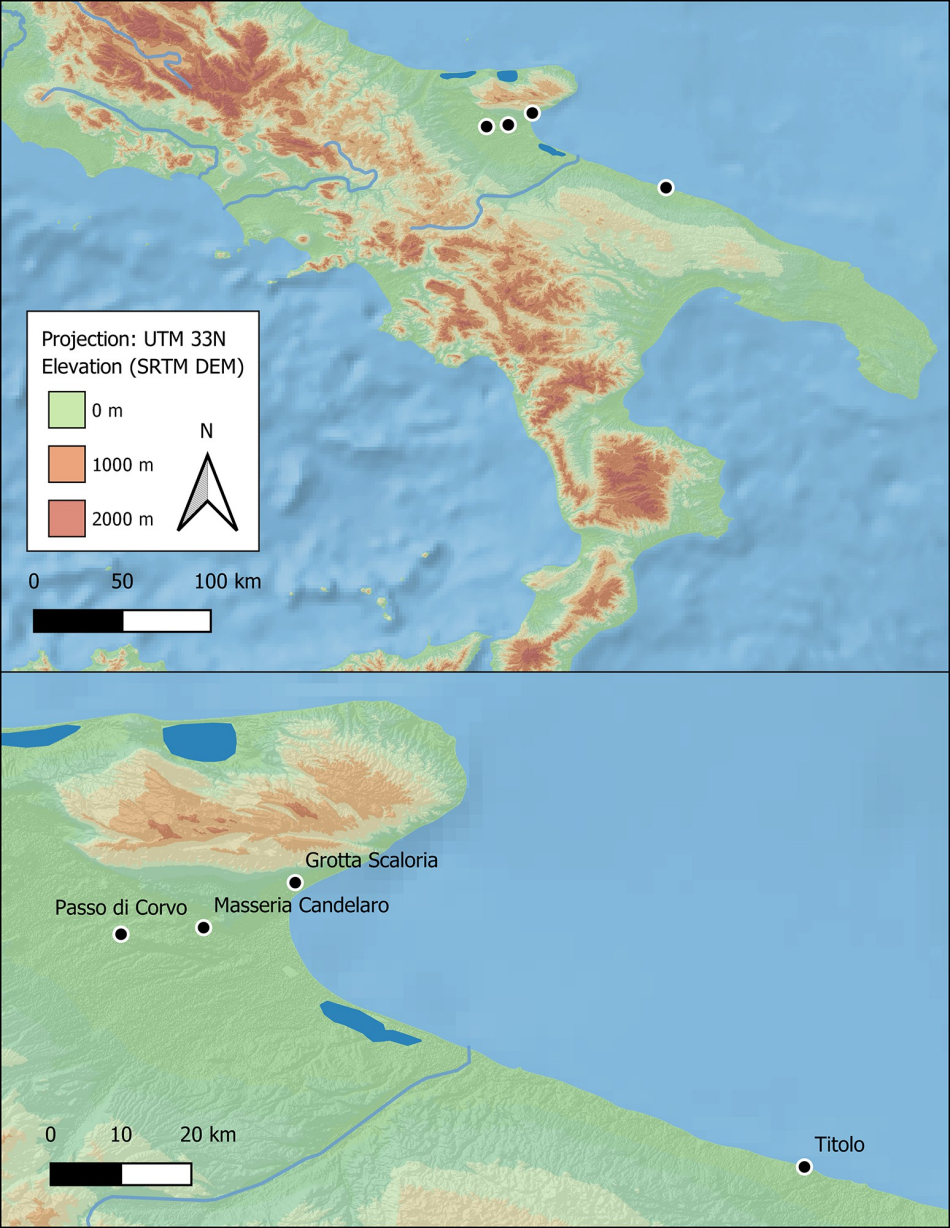
Map of sites studied.

**Fig 2:**
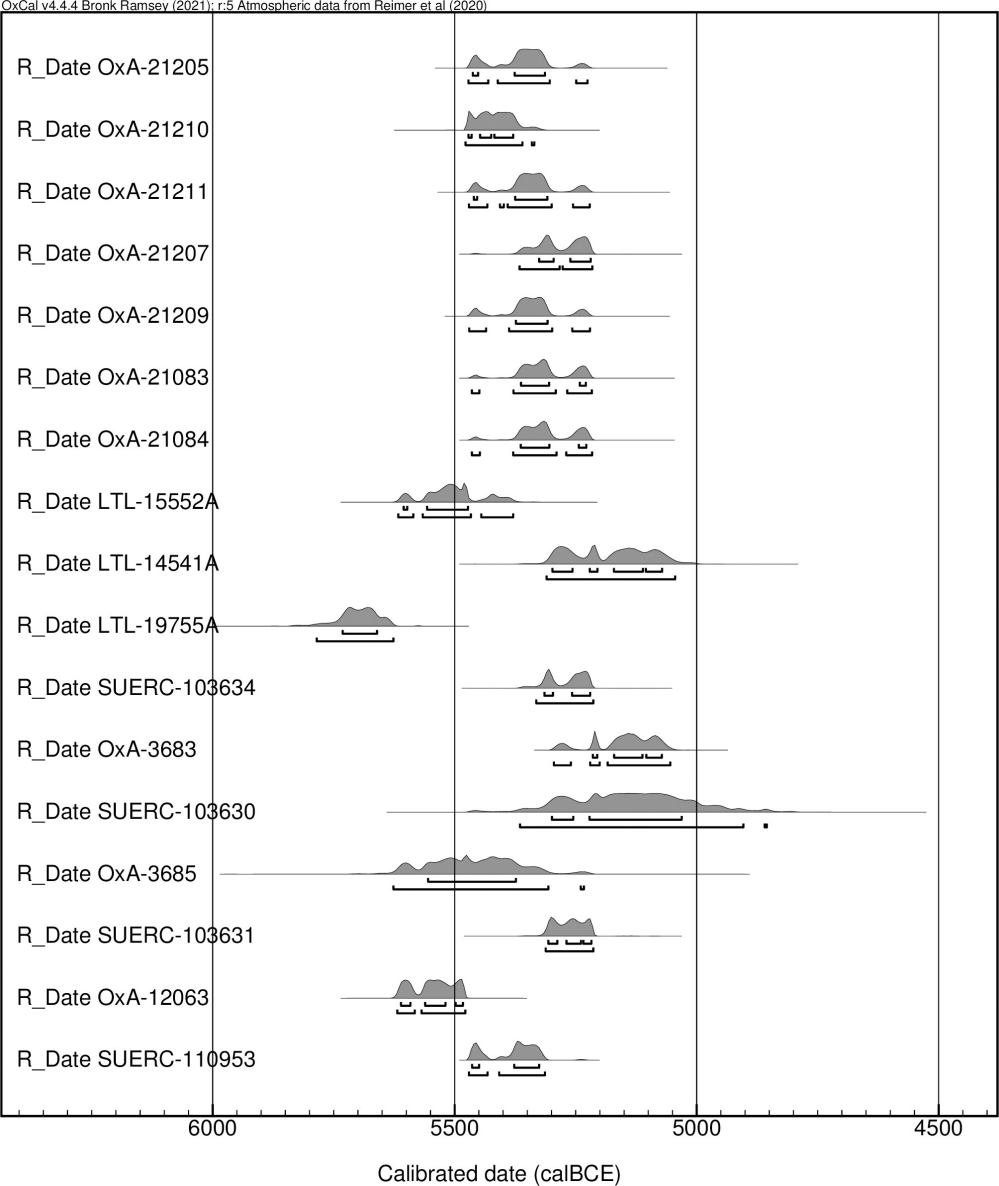
Plot of radiocarbon dates obtained on human remains included in this study (produced in OxCal v.4.4.4).

The remains of 40 human individuals were selected for histotaphonomic analysis from 4 Neolithic sites in Apulia: Grotta Scaloria, Masseria Candelaro and Passo di Corvo (Foggia) and Titolo (Bari) (Fig 1). In Foggia, the sites are located within a radius of ca. 30 km, while Titolo is ca. 100 km to the south. These sites were predominantly occupied during the Early (6000–5500 cal BCE) and Middle (5500–4300 cal BCE) Neolithic and, in the same periods, utilised for burial and disposal of the dead [60–62]. Seventeen of the individuals included in this study have directly associated radiocarbon dates, and several of these are published here for the first time (Fig 2).

**Fig 3:**
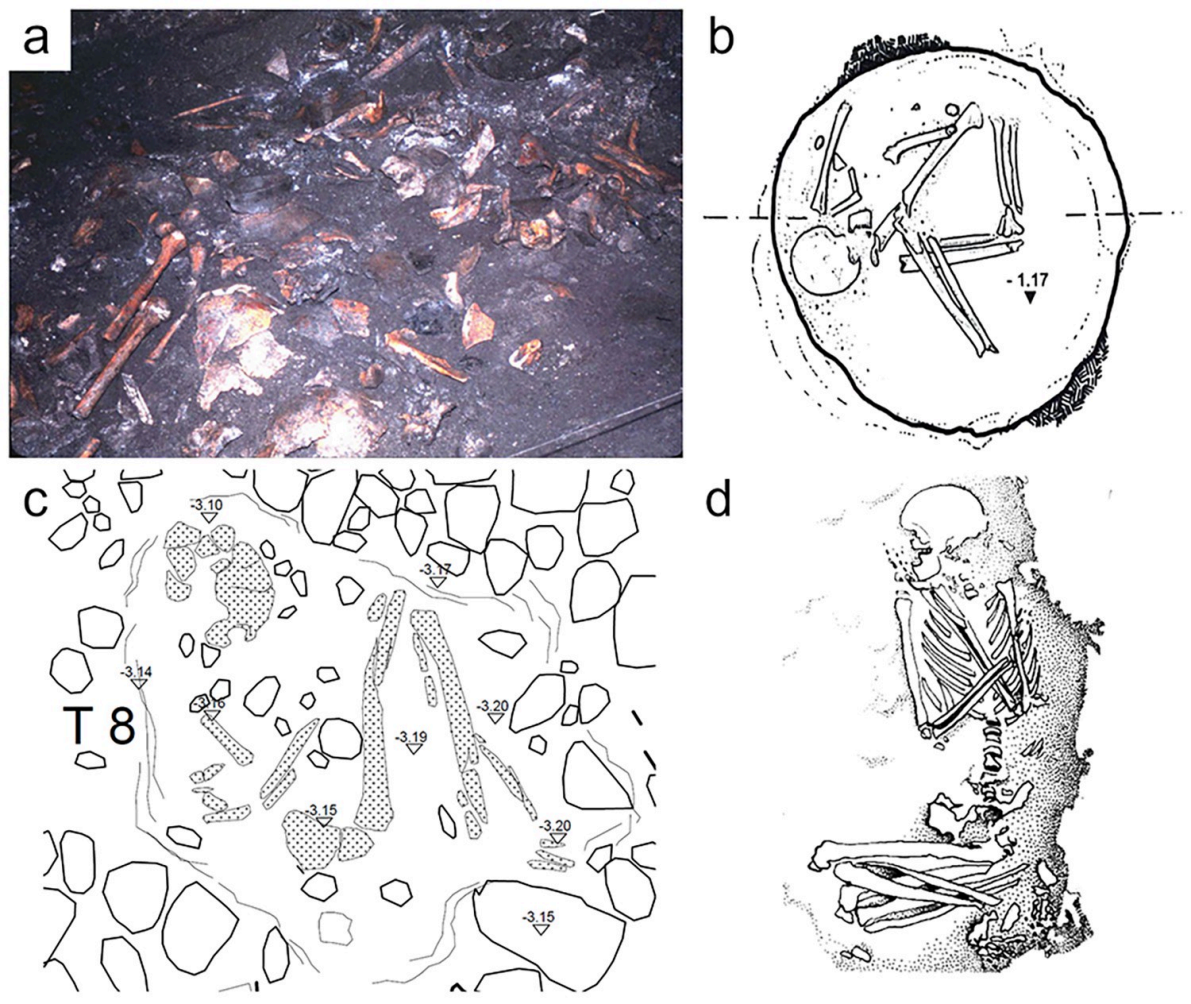
Varied depositions at each site. A) Disarticulated remains in Trench 10 at Grotta Scaloria (photo: S. Winn, reprinted from [72] under a CC BY license, with permission from the Cotsen Institute of Archaeology Press, original copyright 2016); B) Semi-articulated skeleton in pit 40D T1, Masseria Candelaro reprinted from [68] under a CC BY license, with permission from Dr. Italo Muntoni, original copyright 2004; C) Titolo T8 poorly preserved articulated skeleton (illustration by Francesco Sanseverino and Francesco Carbonara published under a CC BY license); D) Passo di Corvo T5 articulated skeleton with hyperflexed lower limbs, reprinted from [69], with permission from Dr. Eugenia Isetti, original copyright 1983.

At each site, funerary treatment and disposal of the dead varied (Fig 3). In Grotta Scaloria, disarticulated human remains were strewn on the cave surface alongside processed animal bones and other occupational debris [63]. Cutmarks on c.6% of the analysed human bone assemblage indicates that body parts were sometimes defleshed, although it is unknown whether they were defleshed after decomposing *in situ* in the cave, or after they had been moved into the cave from elsewhere [64]. There is also evidence of articulated single burials, mostly dating or culturally affiliated to a slightly later period than the commingled remains [63, 65, 66]. In the ditched villages of Masseria Candelaro and Passo di Corvo, burials were made within the boundaries of the village in simple pit graves or disused storage features, as well as in the village ditches [67, 68]. At Masseria Candelaro, numerous disarticulated bones and some cremated fragments were also sporadically deposited in pits and on occupational surfaces [67, 69]. At Passo di Corvo, secondary deposits of disarticulated bone are present, as well as evidence of defleshing in the form of chop- and cutmarks on articulated skeletons and isolated bones [70, 71]. Burials at Titolo were much simpler. Single individuals were placed in shallow pit graves with thin coverings of soil and stone slabs; only one feature contained the partial remains of three individuals [11, 62].

These varied depositional and funerary processes are broadly representative of the known range of treatments of the dead during the 6^th^ and first half of the 5^th^ millennia BCE [1, 7] in central and southern Italy. How these different processes were connected, in terms of a sequence of post-mortem interactions and rites, deserves further exploration. Histotaphonomic analysis provides the opportunity to investigate whether primary burials in varied positions—for example, hyperflexed, flexed, or semi-articulated—display different signatures of microstructural preservation, and to compare the extent of bacterial bioerosion between articulated and disarticulated bones.

The geology of Apulia is largely homogeneous, comprising a karstic landscape of limestone and dolomite platforms, separated by the Tavoliere, Ofanto and Taranto-Brindisi plains [73]. Karstic activity has produced frequent underground caves and cavities, covered by residual *terra rossa* soils, particularly in the Gargano region [74]. Grotta Scaloria is located in the Gargano on Mesozoic-Cenozoic carbonate deposits. Passo di Corvo and Masseria Candelaro are located at the margins of the alluvial basin of the Tavoliere, comprising Quaternary deposits of silty-clay or sandy grey soils, overlying Pleistocene and Plio-Pleistocene gravel and sand deposits [75, 76]. Chemical analysis of the alluvial soils of the Tavoliere indicated that iron oxides comprise between around 4–5 wt% of the sandy grey soils, increasing to 7–8 wt% in the silty-clay deposits [77]. Titolo is situated on the limestone *Calcare di Bari* outcrop on the coastline. The soils in these regions, when subjected to cycles of precipitation and aridity, are prone to producing concretions which can cover and adhere to bone surfaces. Burials and depositions at Masseria Candelaro, Passo di Corvo and Titolo were made either in the soil, in cavities cut into the limestone, or in sub-surface storage pits or wells which were either backfilled or exposed and gradually infiltrated by soils. At Grotta Scaloria, depositions were made on the clay surface of the Upper Cave and bones were exposed to the microclimate within the cave, including elevated humidity, periodic inundation, and speleothem formations [78]. All burials were made in aerobic environments, usually in temperate conditions and in well-draining soils.

## 3. Materials and methods

Forty individuals from a range of depositional contexts and states of articulation were chosen for study, including several individuals or bone fragments which presented cutmarks (Table 1). Samples were categorised according to three states of funerary treatment through reference to excavation photographs and descriptions: articulated skeletons (where the *in situ* presence of anatomical connections was observed), semi-articulated remains (where a mixture of articulated, loose, and disarticulated joints were observed), or disarticulated remains (see Fig 3 for examples). This allowed us to investigate post-mortem histories at a histological level in tandem with macroscopic taphonomic analysis. Many individuals from these sites have been the subject of multi-isotopic analyses investigating dietary practices and residential mobility [79–83]. For 29 of the individuals included in this study, corresponding ^87^Sr/^86^Sr isotopic measurements from bone and/or enamel were available [82, 83] (S1 File). These provide a proxy of geological origins and later life locality [84, 85]. From these, we have investigated the relationship between bacterial attack (as a proxy for early post-mortem funerary treatment) and individual mobility. Titolo is excluded from this analysis because isotopic measurements from these individuals are not yet published. The relationship between lifetime mobility and histotaphonomic preservation has not been directly investigated in previous studies and represents a novel approach to funerary treatment.

**Table 1:** Archaeological context, radiocarbon date, skeletal element, osteological age and sex, and state of articulation for all histological samples.

Site	Sample ID	Context	Date (BP)	Element	Age	Sex	Deposition
Grotta Scaloria	GS2	Tr 10. Burial group 2, ’A’ indiv		Femur	Nonadult	ND	Disarticulated
	GS5	SC78, H8, L16		Femur	Adult	ND	Disarticulated
	GS6	SC78		Femur	Adult	ND	Disarticulated
	GS9	Tr 6. L1		Femur	Adult	ND	Disarticulated
	GS13	Tr 10 ’floor of graves’		Femur	Adult	ND	Disarticulated
	GS14	Tr 10. ’Homo’		Femur	Adult	ND	Disarticulated
	GS16	Tr 10. Burial group 1, ’A’ indiv	OxA-21205: 6381±33	Femur	Nonadult	ND	Disarticulated
	GS19	Tr 10. Burial group 2, ’C’ indiv		Femur	Nonadult	ND	Disarticulated
	GS20	Tr 10. Burial Group 2, ’C’ indiv		Femur	Nonadult	ND	Disarticulated
	GS21	Tr 10. Burial Group 2, ’C’ indiv	OxA-21207: 6324±32	Femur	Adult	ND	Disarticulated; cutmarks
	GS22	Tr 10. Burial group 3, ’B’ indiv		Femur	Nonadult	ND	Disarticulated
	GS25	Tr 10. Burial Group 5, ’A’ indiv	OxA-21209: 6368±31	Femur	Nonadult	ND	Disarticulated
	GS26	Tr 10. Burial Group 5, ’B’ indiv		Femur	Nonadult	ND	Disarticulated
	GS28	Tr 10. Burial group 6, ’A’ indiv	OxA-21210: 6448±31	Femur	Adult	ND	Disarticulated
	GS31	Tr 10. Burial group 8, ’A’ indiv	OxA-21211: 6371±33	Femur	Nonadult	ND	Disarticulated
	GS36	Tr 10. Burial Group 9, ’B’ indiv	OxA-21083: 6348±31	Femur	Adult	ND	Disarticulated
	GS40	Tr 10E, L2		Femur	Adult	ND	Disarticulated
	GS41	Tr 10E, L2	OxA-21084: 6347±32	Femur	Adult	ND	Disarticulated
Titolo	PAL015	T1		Femur	Adult	ND	Semi-articulated (poorly preserved)
	PAL011	T2	LTL-15552A: 6541±50	Femur	25–35	?M	Semi-articulated (possibly empty space)
	PAL013	T6	LTL-14541A: 6230±50	Femur	35–45	M	Semi-articulated (possibly empty space)
	PAL012	T8	GU61653: Failed	Femur	35–45	?F	Articulated
	PAL014	T9	LTL-19755A: 6815±45	Femur	35–45	F	Semi-articulated (possibly empty space)
Masseria Candelaro	CND019	40D T1 89	SUERC-103634: 6314±22	Femur	45–60	?M	Semi-articulated
	CND020	Ditch F T1	OxA-3683: 6212±24	Femur	25–45	F	Articulated
	CND021	Ditch F T2		Femur	45–60	F	Semi-articulated (possibly empty space)
	CND022	Ditch F T3	SUERC-103630: 6200±95	Femur	35–45	M	Articulated
	CND023	Ditch F, T4A		Femur	25–35	?F	Disarticulated
	CND024	Ditch F, T4B	OxA-3685: 6510±95	Femur	25–45	?M	Disarticulated
	CND025	P3/4A		Femur	12–18	ND	Articulated; prone
	CND026	P3/4B	SUERC-103631: 6289±25	Femur	45–60	F	Articulated
	CND027	T40 D P2 8	SUERC-103631: 6601±37	Femur	35–45	F	Articulated
	CND028	P2 8 base		Femur	Adult	ND	Disarticulated
	CND029	Fa 5		Femur	Adult	ND	Disarticulated
Passo di Corvo	PDC001	T7	SUERC-110953: 6398±22	Femur	Adult	?M	Disarticulated; cutmarks on some elements
	PDC002	T5		Fibula	Adult	M	Articulated; femoro-tibial joints hyperflexed
	PDC004	T12	GU64339: Failed	Long bone	Old child	ND	Disarticulated
	PDC005	T3		Tibia	6–7 yo	ND	Articulated; cutmarks on some elements
	PDC006	T9a	GU64338: Failed	Femur	14–16 yo	?F	Disarticulated; cutmarks
	PDC007	T11		Femur	Adult	ND	Articulated; prone

Note that Tr = Trench; T = *tomba*/grave number; P = *pozzo*/pit number, ND = sex not determinable. Data on osteological age and sex from the Grotta Scaloria samples obtained from [90]; for the Passo di Corvo samples obtained from [70]; for the Masseria Candelaro and Titolo samples, determined by authors, although also see [69] for previous analysis of Masseria Candelaro skeletal remains. Radiocarbon dates obtained from [60–62, 91, 92], date from PDC001 first published here.

Small samples (from 1–2 cm in maximum dimension) of long bone, preferably the femur, were cut using a handheld Dremel rotary drill saw. Bone sections were impregnated in a mixture of crystic polyester resin, acetone and Methyl Ethyl Ketone, and subsequently cured in a vacuum chamber and a drying oven until hardened. Samples were then sliced into transverse sections, mounted on glass slides, and ground to approximately 40–50 μm (as appropriate for each batch of samples). Undecalcifed thin sections were studied using a Leitz Laborlux 12 Pol S microscope, under normal and polarised transmitted light, with lenses providing x50, x100 and x250 magnification. A GXCAM-U3PRO 20MP camera was used for imaging.

Each thin section was assessed according to several histological indices under transmitted and polarised optical light microscopy. The Oxford Histological Index (OHI) semi-quantitatively scores the extent of bone which is affected by microbial micro-foci of destruction (or non-Wedl MFD), from 0 (<5% of the bone is well-preserved) to 5 (>95% of the bone is well-preserved) [86, 87]. The General Histological Index (GHI) uses the same scale for scoring as the OHI, but takes into account the amount of bone which is damaged by non-Wedl MFD as well as Wedl MFD and other diagenetic factors such as cracking and staining [28]. Cracking, staining, and inclusions were noted as present or absent and qualitatively described [14]. A distinction was observed between dark and light coloured microcracks in the samples, which we suggest reflects ancient cracking versus microcracks produced during cutting or sample preparation, respectively. Collagen birefringence was assessed under polarised light and scored using the Birefringence Index (BI), with birefringence characterised as either obliterated, reduced, or normal [88]. Each sample differed in size available for analysis once mounted on slides. When bone histological preservation was observed to vary across the available sample, scores were assigned as an average. Each sample was scored by one observer (JET) and most were scored by a further observer (TB), with the final scores agreed by consensus. Statistical analysis was carried out in R Studio v.4.3.3 and charts were produced using the R package “ggplot2” [89].

### 3.1 Ethics of destructive analysis of ancient human remains

Histological analysis is destructive to the sampled human bone, requiring at minimum 1x1 cm full-thickness cortical samples. This research received approval from the Ethics Committee of the Department of Archaeology, University of Cambridge and followed internal project guidelines, including the full recording of remains prior to removing samples. To ensure minimal destruction, the majority of samples included in this study originated from larger samples which had already been cut for prior studies. All necessary permits were obtained for the described study, which complied with all relevant regulations. The Soprintendenza Archeologia, Belle Arti e Paesaggio per le province di Barletta-Andria-Trani e Foggia authorised this research on the human remains from Grotta Scaloria (stored at the Department of Archaeology, Cambridge, UK), Masseria Candelaro (stored at the Museo delle Civiltà, Rome, Italy), and Passo di Corvo (stored at the University of Bologna and Sapienza University, Italy). The human remains from Titolo are on loan to the Museo Giuseppe Sergi (Sapienza University, Rome) and their analysis was approved by the Soprintendenza Archeologia, Belle Arti e Paesaggio per la Città Metropolitana di Bari.

## 4. Results

**Fig 4:**
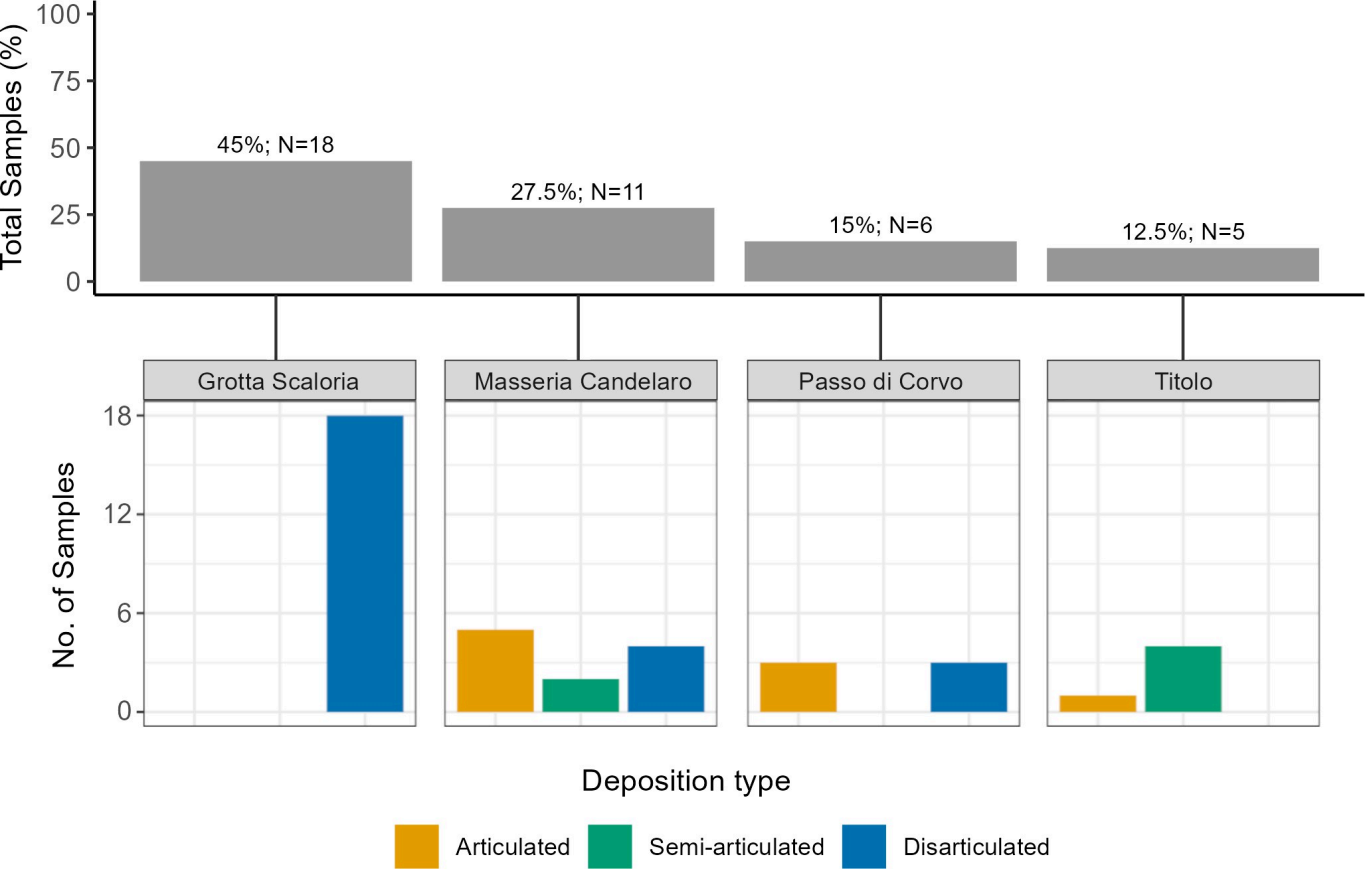
Top: Percentage and number of samples from each site; Bottom: Samples from each site categorised according to state of articulation.

All of the individuals sampled from Grotta Scaloria were disarticulated; from Passo di Corvo, remains were either articulated or disarticulated; at Titolo, samples were retrieved from skeletons which were semi- or fully articulated, and at Masseria Candelaro samples were retrieved from individuals in all states of articulation (Fig 4). Overall, 22.5% of samples were obtained from articulated skeletons, 15% from semi-articulated individuals, and 62.5% from disarticulated bones.

**Fig 5:**
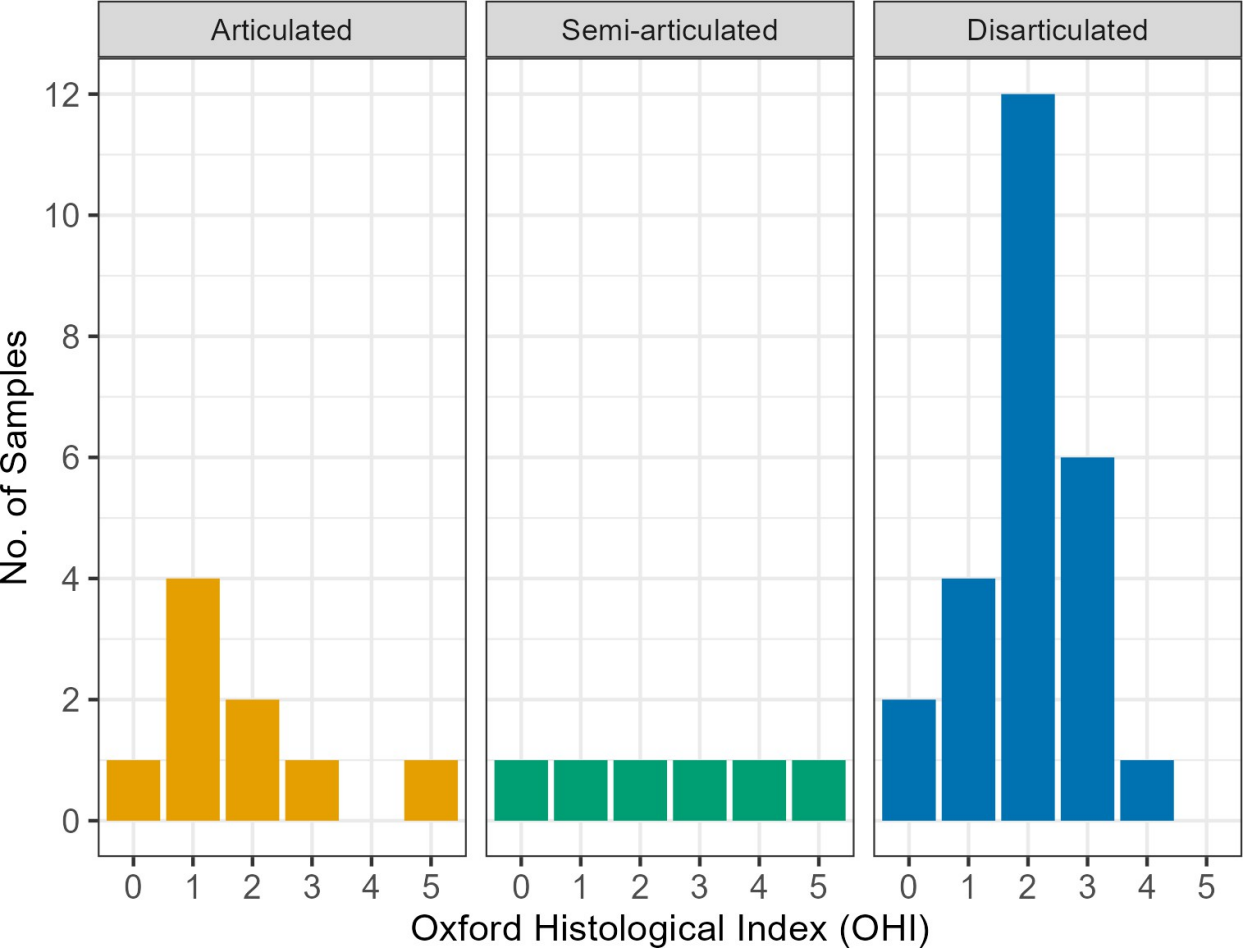
Plot of Oxford Histological Index (OHI) scores according to state of articulation.

Qualitative and semi-quantitative results of the histotaphonomic analysis are outlined in Table 2 and micrographs of each sample are available in S2 File. Patterns of bacterial bioerosion were variable across sites and sometimes even in remains from the same context, despite all having originated from similar depositional environments. Of the 40 samples, 1 was ground marginally thinner than required (GS5) and while it has been scored, it should be noted that the whole sample was not observable. Overall, 32.5% (n = 13) of samples displayed extensive bacterial bioerosion (scores 0–1), 32.5% (n = 15) displayed moderate bacterial bioerosion (score 2), and 25% (n = 10) showed arrested bacterial bioerosion (scores 3–4). Only 2 samples (PDC007, PAL014) were free of bacterial bioerosion and displayed well-preserved bone microstructure (5%). When analysed according to articulation, the extent of bacterial bioerosion varied widely across articulated and semi-articulated individuals (with scores from 0–5), including two individuals without evidence of bacterial bioerosion (Fig 5). Disarticulated bones presented OHI scores from 0–4, with the majority presenting bacterial bioerosion over >50% of the bone.

**Table 2:** Results of histotaphonomic analysis.

Site	Sample ID	Local?	OHI	GHI	BI	Cracking	Staining	Inclusions	Wedl MFD	Comments
Grotta Scaloria	GS9	Local	1	1	1	Slight	Slight orange-coloured infiltrations	Orange-red coloured inclusions and infiltrations	Yes	
	GS2	Local	2	1	0.5	Moderate			Yes	
	GS13	Local	3	2	0.5	Slight	None	None	None	
	GS16	Non-local	3	2	0.5	Slight	None	None	None	
	GS28	Non-local	2	2	0.5	Slight	None	Some orange-coloured concretions adhering to trabeculae	Yes	
	GS19	Local	2	1	0.5	Slight	None	None	Yes	
	GS22	Local	2	1	0.5	None	Orange-coloured staining along periosteal margin	Slight concretion on periosteal margin	None	
	GS31	Local	2	1	0.5	Slight	None	Framboidal inclusions adhering to trabeculae	Yes	
	GS5	Local	3	-	-	-	-	-	-	Sample ground too thin to observe fully
	GS6	Local	1	1	0	Slight	None	None	None	
	GS14	Local	3	2	0.5	Moderate	None	None	None	
	GS20	Local	2	1	0.5	Slight	None	Slight concretions on trabeculae and/or within Haversian canals	None	
	GS21	Local	4	3	0.5	Slight	None		None	
	GS25	Non-local	2	1	0.5	Slight	None		None	
	GS26	Local	2	2	0.5	None	None	Concretions on periosteal and endosteal surfaces	None	
	GS36	-	0	1	0	Slight	None	Small orange framboidal inclusions; concretion within bone and on periosteal surface	None	
	GS40	Local	2	2	0.5	Slight	None	Orange-coloured framboidal inclusions and discoloured concretion on periosteal and endosteal margins	None	
	GS41	Local	3	3	0.5	None	None		None	
Titolo	PAL011	-	0	0	0	None	Orangey-red coloured infiltrations	Concretion with red discolouration on periosteal margin	None	
	PAL013	-	2	1	0	Extensive	None	Concretion on periosteal and endosteal surfaces with red discolouration; framboidal inclusions adhering to trabeculae	None	
	PAL012	-	2	2	0.5	Slight	Slight reddish-brown staining	Framboidal inclusions adhering to trabeculae	Yes	
	PAL014	-	5	4	0	Extensive		Red discolouration to concretions on periosteal and endosteal surfaces	None	
	PAL015	-	1	1	0.5	None	None	None	None	
Masseria Candelaro	CND019	Non-local	3	2	0.5	Moderate	Slight reddish-brown staining	Small orange framboidal inclusions	Yes	
	CND020	Non-local	1	1	0	Slight		Concretions on periosteal and endosteal surfaces and/or within Haversian canals, occasionally with red discolouration	Yes	
	CND021	Non-local	4	3	0	Slight	None		Yes	Rare organic matter, possibly fungal
	CND022	Local	3	2	0.5	Slight	Slight reddish-brown staining		None	
	CND023	Local	3	2	0.5	Slight			None	
	CND024	Local	3	2	0.5	Slight			Yes	
	CND025	Local	2	2	0.5	Slight	Reddish-brown staining across most of sample		None	
	CND026	Local	1	1	0.5	Moderate	Slight reddish-brown staining		None	
	CND027	Local	0	0	0	None	None		None	
	CND028	Local	1	1	0	Slight	Reddish-brown staining across most of sample		None	
	CND029	-	2	2	0.5	Slight			None	
Passo di Corvo	PDC001	Local	2	1	0.5	Moderate	Slight reddish-brown staining with infiltrations in Haversian canals	None	Yes	Rare organic matter, possibly fungal
	PDC002		1	1	0	Moderate		Concretions on periosteal and endosteal surfaces; dark brown inclusions within some Haversian canals	No	Rare organic matter, possibly fungal
	PDC004		1	1	0	Moderate		Concretions on periosteal and endosteal surfaces and/or within Haversian canals, occasionally with red discolouration	No	
	PDC005		1	1	0.5	Moderate			Possible	
	PDC006		0	0	0	None	None		None	
	PDC007	Local	5	4	0	Extensive	None		Yes	

**Fig 6:**
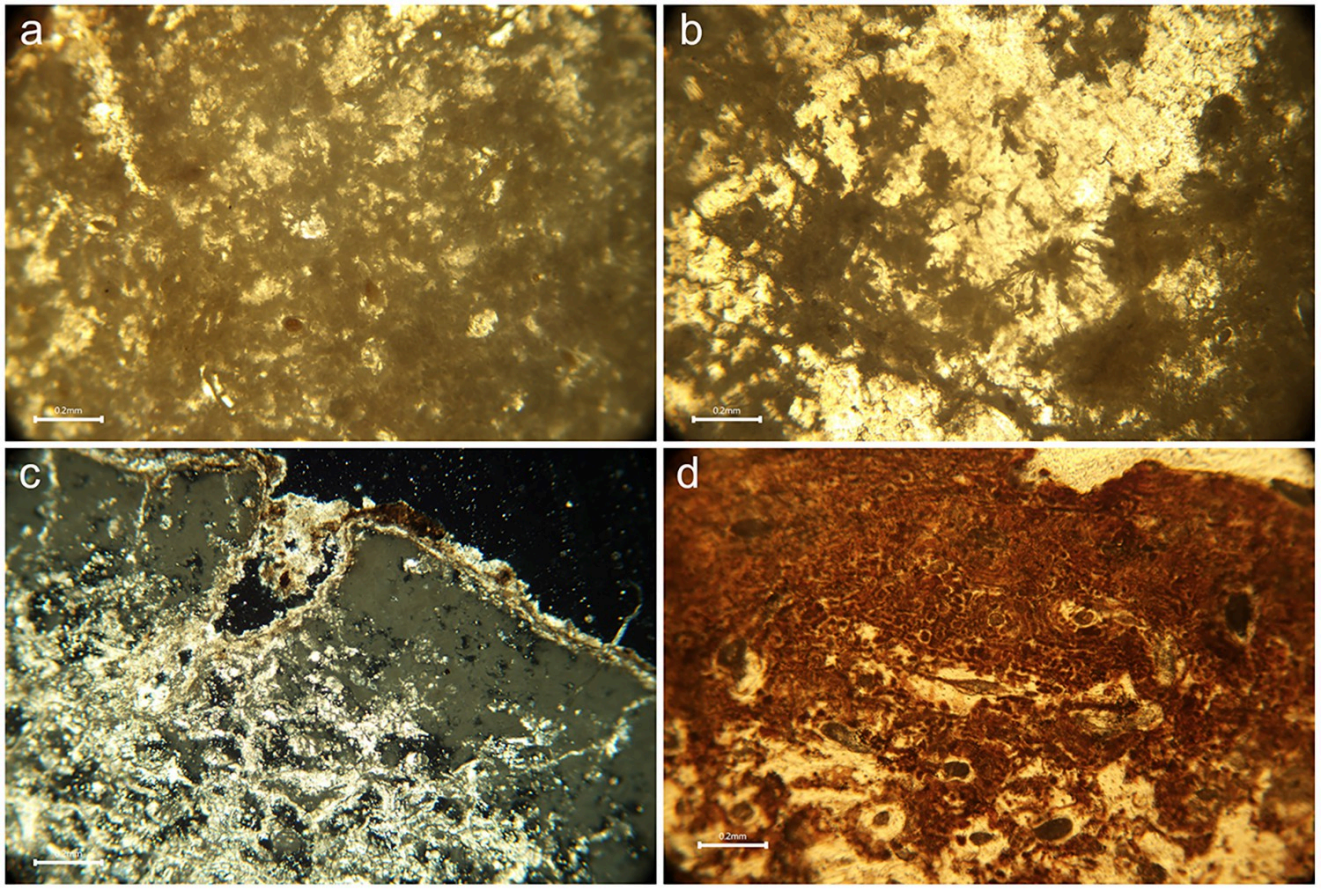
Micrographs of bone thin sections illustrating various histotaphonomic characteristics. a) Amalgamations of non-Wedl micro-foci of destruction (MFD) in sample PAL011 which appear as dark ‘cloudy’ masses of erosion surrounding the osteons, and stained infiltrations in some Haversian canals; b) Wedl MFD in sample GS19 observed as dark and meandering erosive defects, some of which emanate from cracks in the bottom left quarter of the image; c) Inclusions in sample GS36 which are identified as bright (birefringent) white and golden coloured structures on the external/periosteal surface of the bone (from top left to middle right) and within the micropores and cracks in the mesosteal third of the bone (middle to bottom left) under polarised light; d) Probable iron oxide staining in sample CND025 presents as mosaic-like formations of dark reddish-brown intrusions, distributed thickly along the periosteal band and more sparsely in the mesosteal third of the sample (bottom half of the image). All figures author’s own, published under CC BY license.

General histological damage (GHI) was usually consistent with the OHI scores (n = 26), or slightly poorer, reflecting the presence of probable Wedl MFD and/or cracking, staining or inclusions. Several samples display extensive alteration (GS40, PAL011, CND020, CND027, CND029), but amalgamations of non-Wedl MFD are clearly observed surrounding osteons. Close comparison for this extensive bacterial bioerosion can be drawn with other archaeological examples (for example, Figs 6F and 7D in [43] and Fig 1 in [47]). However, bacterial bioerosion may be masked by generalised destruction when the internal structure of the bone is extensively altered. Two samples, both from Grotta Scaloria (GS19 and GS22), show some features consistent with generalised destruction. The internal microstructure is greatly altered and the features surrounding osteons are amorphous, obfuscating their clear identification. Future application of bSEM could disentangle the processes responsible for poor bone preservation within these two samples.

Under polarised transmitted light, all samples showed reduced or fully obliterated collagen birefringence, demonstrating widespread collagen loss. Birefringence was absent in areas of the samples which corresponded with the presence of specific non-Wedl MFD or generalised destruction. When bacterial bioerosion was observed, non-Wedl MFD usually could not be distinguished according to the morphological types identified by Hackett [18], and instead represented amalgamations of various forms (Fig 6A). These amalgamations, or coalescences, had typically eroded the bone microstructure along bands of varying thickness on the periosteal and endosteal margins. In extreme cases, bacterial bioerosion was observed to have affected the full cross-section of the bone sample, although occasionally rare islands of several unaffected osteons were present.

On 13 samples, sinuous, transversely-orientated branching defects were observed, which appeared to have invaded the bone along ancient micro-cracks or pervaded from the periosteal band, sometimes in association with bacterial bioerosion (Fig 6B). These were mostly present in samples from Grotta Scaloria (n = 5), but they were also observed on samples from Masseria Candelaro (n = 4), Passo di Corvo (n = 3) and Titolo (n = 1). These defects most closely resemble Wedl MFD; although, as mentioned above, there is ongoing debate about the cause of these phenomena, thus complicating their interpretation [19–22, 37].

Inclusions were observed in most samples, referring predominantly to sediment or calcite concretions on the endosteal and/or periosteal surface. Sometimes, these mineral concretions had pervaded micro-cracks and porous spaces within the bone and were observed within Haversian canals. In one exceptional case (GS36), much of the internal bone microstructure was permeated by mineral inclusions, which were birefringent when observed under polarised light (Fig 6C). This provides further evidence for observations made by the excavators that some bone fragments visibly resembled stalactite pieces [64]. At all sites, evidence of staining was observed, typically of a rusty orange to dark red colour, occasionally alongside framboidal inclusions. Staining was visible on several samples from Masseria Candelaro and Passo di Corvo as bands of reddish-brown discolouration interspersed with bioerosion on the periosteal third of the bone samples (Fig 6D); on CND025, the staining appears to flow into the less bioeroded mesosteal third. On other samples (e.g. CND019, CND025, PDC005, see Figs 47, 59, 75 in S2 File), staining is more diffuse and resembles infiltrations into the bone microstructure, particularly Haversian canals. This is interpreted as iron oxide staining secondary to bioerosion [28], presumably originating from iron-rich soils.

The distribution of cracking, inclusions, and staining according to site, state of articulation, and locality (as inferred from Strontium isotopic measurements) was explored using Fisher’s exact test in R, and the significance level was set at p = 0.05. Cracking was present in the majority of samples from each site (p = 0.5489), depositional mode (p = 0.6021) and in local and non-local individuals (p = 0.5526). Inclusions predominated at all sites and, interestingly, were present on all samples from Masseria Candelaro, principally caused by extensive concretion, although this is not statistically significant (p = 0.2159). According to type of deposition, inclusions were again prevalent across all categories (p = 0.2346) and observed in all articulated remains. Inclusions were present at similarly high levels in local and non-local individuals (p = 1). The prevalence of staining according to site was statistically significant (p = 0.0029), with staining rarely observed on samples from Grotta Scaloria, compared to those from the other sites. When tested according to depositional mode, staining was not significant (p = 0.1034), although it was more frequently observed on samples from articulated and semi-articulated remains. Finally, staining was almost equally observed in samples from local and non-local individuals (p = 1).

### 4.1 Bacterial bioerosion according to archaeological context

**Fig 7:**
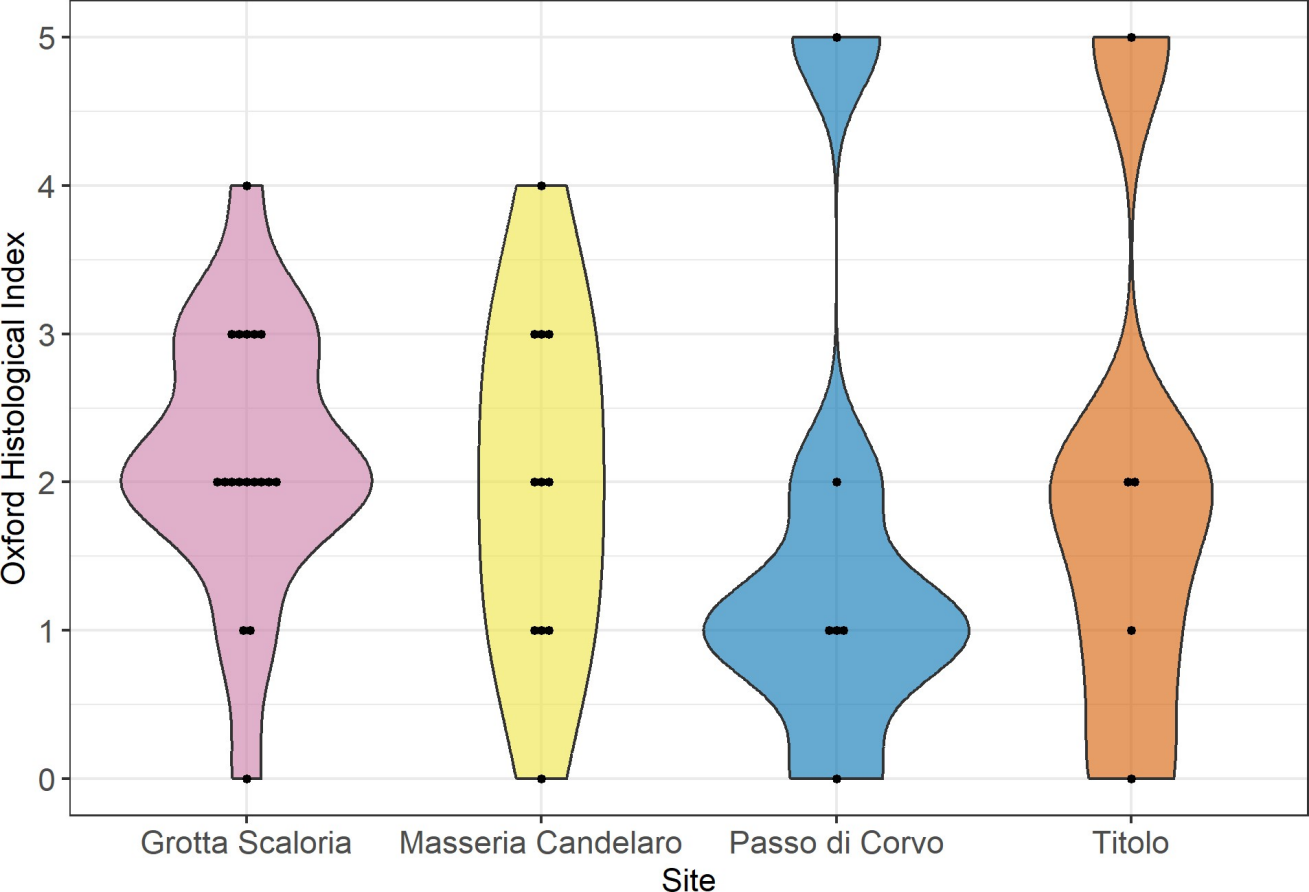
Violin plot of OHI scores according to site; each dot represents a single sample.

Analysed on a site-by-site basis, bacterial bioerosion is variable (Fig 7). At Grotta Scaloria, most samples displayed arrested bacterial bioerosion, with only 3 samples (16.6%) presenting extensive bacterial bioerosion. At Masseria Candelaro, samples were almost evenly distributed across scores from 0–4, reflecting extensive and arrested bacterial bioerosion. At Passo di Corvo and Titolo, similarities are evident, with a bimodal distribution of predominantly extensive or arrested bacterial bioerosion (scores 0–2), and one sample at each site with no bioerosion. A Kruskal-Wallis test determined that bacterial bioerosion was not statistically different according to site (H(3) = 2.29767, p = 0.5131).

Previous isotopic and taphonomic research on the commingled remains from Grotta Scaloria suggested that cutmarks may have been more prevalent on local individuals, based on ^87^Sr/^86^Sr measurements from bone [79]. Including available isotopic data from Masseria Candelaro and Passo di Corvo [82], the relationship between bacterial bioerosion and inferred locality was analysed. Given the variation in the isotopic data, in part related to the differential availability of suitable bones and teeth for analysis, locality has been assessed differently at each site. Since most remains were disarticulated at Grotta Scaloria, only the direct ^87^Sr/^86^Sr measurements on the same bones used for this study could be considered, representing an average adulthood signature of geological location. However, we are aware that ^87^Sr/^86^Sr measurements from bone and dentine are more susceptible to diagenesis and the results are therefore interpreted with caution [85]. For most of the individuals considered from Masseria Candelaro and Passo di Corvo, ^87^Sr/^86^Sr measurements on bone were also supplemented by ^87^Sr/^86^Sr measurements on tooth enamel; in these cases, locality was assessed based on childhood signatures from the enamel (S1 File). A Mann-Whitney U test found that OHI scores were *not* significantly different based on origin (local or non-local): U = 57.5, p = 0.4474. This suggests that early post-mortem funerary treatments may not have differed according to an individual’s childhood place of origin or adulthood locality.

#### 4.1.1 Bacterial bioerosion in disarticulated bones

Arrested bacterial bioerosion (OHI scores 3–4) was mostly observed on the disarticulated bones from Grotta Scaloria, alongside several samples from disarticulated, articulated and semi-articulated remains from Masseria Candelaro. Arrested bacterial bioerosion has previously been encountered on partially articulated skeletal remains [54], disarticulated bones from disturbed burials in chamber tombs [43] and disturbed primary burials [57]. In these studies, however, scores of 3 (indicating 51–85% of bone free of bacterial bioerosion) were rare or absent, whereas they were encountered regularly in the Grotta Scaloria and Masseria Candelaro remains. At Titolo and Passo di Corvo, where most samples derived from straightforward primary burials, only 3 samples across both sites scored 2, which may be within the range of variation seen for bones buried in aerobic soils [38].

Analysis of bacterial bioerosion in the disarticulated remains from Grotta Scaloria, Masseria Candelaro and Passo di Corvo provides the opportunity to investigate which primary contexts these bones may have originated from and the types of funerary treatment they may have been subjected to prior to deposition. Disarticulated bones and fragments predominantly showed that bacterial bioerosion affected either more or less than 50% of the observable sample. In contrast, samples from articulated remains mostly showed that bacterial bioerosion affected 85% (or more) of the bone microstructure. However, several samples from articulated and semi-articulated burials at Titolo and Masseria Candelaro presented similar extents of bacterial attack as the disarticulated remains (including Titolo T6 and T8 and Masseria Candelaro T3 and P3/4A).

#### 4.1.2 Intra-feature variation in bacterial bioerosion at Masseria Candelaro

The large number of human remains excavated at Masseria Candelaro, including contexts containing multiple individuals who were often in different states of completeness and articulation, provided the opportunity to compare histotaphonomic features from remains within the same context or area of the site. Samples from two burials at the base of a silo, P3/4A, an adolescent in a prone position, and P3/4B, an old adult female in a flexed position, presented slightly different histotaphonomic features. Given their deposition in a relatively small space (c. 0.65 m diameter) at the bottom of a 1.65 m depth of stratigraphy, it can be presumed that they were deposited simultaneously or in short sequence [67]. They presented extensive to moderate bacterial bioerosion (P3/4A OHI = 1, P3/4B OHI = 2), focused predominantly on the periosteal band, with reduced collagen birefringence, consistent with burial. However, P3/4B displayed rusty, red-coloured staining in the bioeroded zone, probably from iron oxides, while P3/4A was free of staining. This may suggest that they were buried at slightly different times, or that one of the bodies received a short period of pre-burial treatment in a different location.

Another silo, P2 8, contained an articulated skeleton of an old-middle aged female placed on their right side with their upper body extended and lower legs flexed [67]. They were deposited near the base of the feature, with disarticulated human bones also distributed in underlying and overlying levels, alongside a large assemblage of ceramic sherds, faunal bones, lithic and bone tools, and various cereal, plant, and floral seeds. The sample from the skeleton showed full bacterial bioerosion (OHI = 0) with obliterated collagen birefringence. This suggests that the body was rapidly buried and decomposition was not interrupted. A disarticulated fragment of femur at the base of the pit (sample CND028) showed extensive bacterial attack (OHI = 1), but islands of one or several unaffected osteons were present; collagen birefringence was obliterated except for in these areas, where it was reduced. This might suggest that the bone was removed from another primary burial of an individual who had already undergone decomposition and burial.

**Fig 8:**
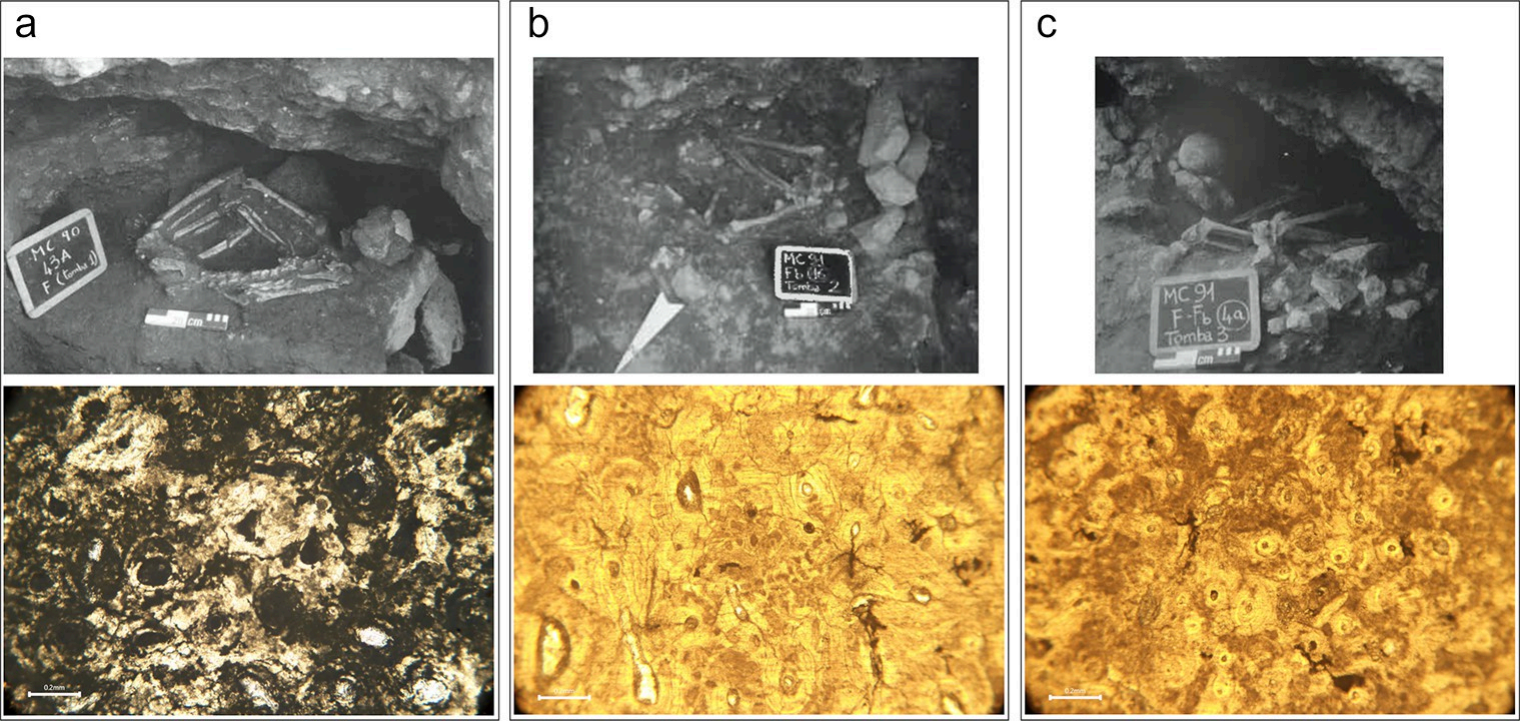
Masseria Candelaro Ditch F burials with excavation photographs on top row (reprinted from [59] under a CC BY license with permission from Dr. Italo Muntoni, original copyright 2004) and micrographs under normal transmitted light below; a) T1 (CND020), micrograph shows bacterial bioerosion surrounding osteons; b) T2 (CND021), micrograph shows bone is mostly well-preserved, except for some minor bacterial bioerosion, cracking and Wedl MFD (middle right); c) T3 (CND022), micrograph shows areas of bacterial bioerosion distributed irregularly across the sample.

Finally, each of the three primary burials in the inner ditch presented a different histotaphonomic profile. Radiocarbon dates from two of these indicate that they were buried around the final quarter of the 6^th^ millennium BC [91]. Each was placed in a small cavity cut into the limestone bedrock in the inner faces of the ditch. T1 (CND020, a middle aged female) was placed on their side with their legs hyperflexed (Fig 8A); T2 (CND021, an old adult female) was found in a sitting position with their legs contracted and subsequently disarticulated in the open burial space (Fig 8B); and T3 (CND022, a middle aged male) was placed with their trunk supine and legs flexed on the right side (Fig 8C) [67]. Bacterial bioerosion was extensive in T1 (OHI = 1), consistent with burial in a closed environment soon after death. In contrast, bacterial bioerosion was arrested in both T2 and T3 (OHI = 3) and T2 presents rare Wedl MFD. It is possible that these individuals were deposited soon after death, but the niches remained open for some time, creating the opportunity for invertebrates to scavenge the soft tissues and fungal spores to colonise the bones.

#### 4.1.3 Absence of bacterial bioerosion in two individuals

**Fig 9:**
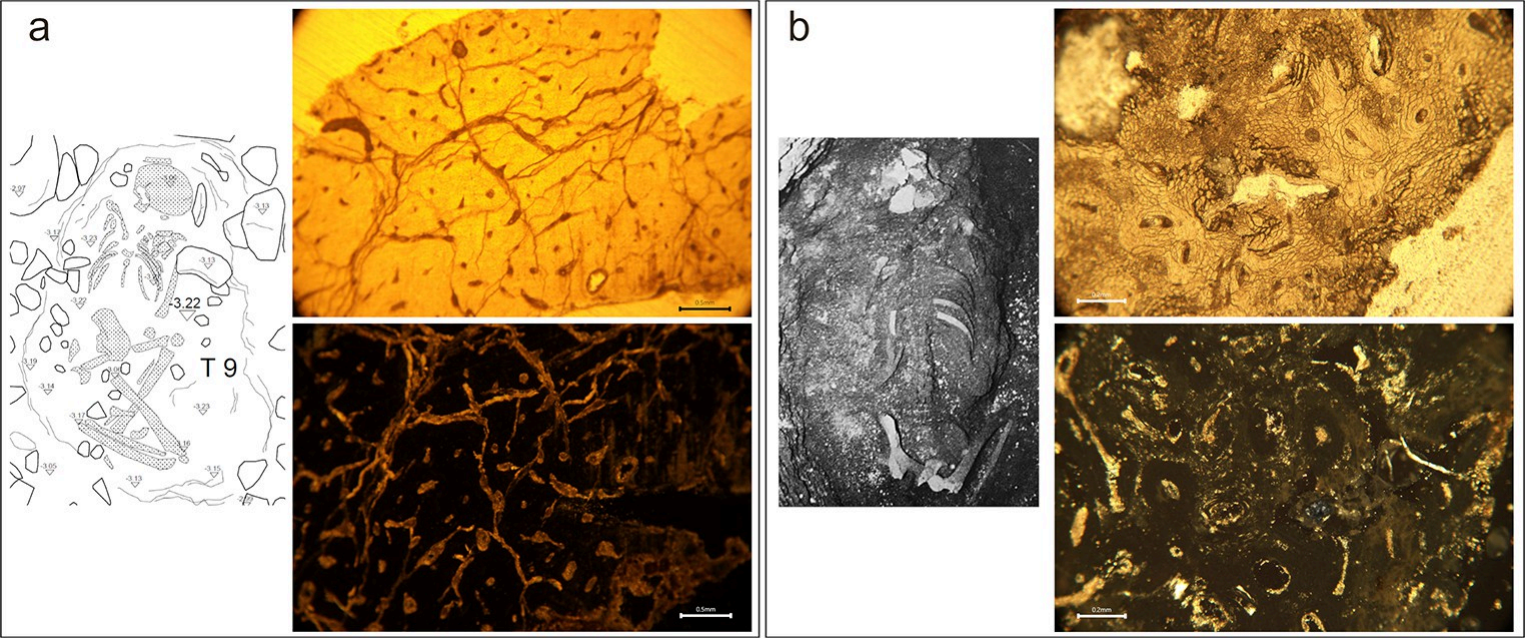
a) Excavation plan of Titolo T9, by Francesco Sanseverino and Giuseppe Carbonara, published under a CC BY license. Micrograph (PAL014) under normal transmitted light (top) showing no bacterial bioerosion but extensive cracking, and under polarised transmitted light (bottom) with no collagen birefringence; b) Photograph of Passo di Corvo T11 (reprinted from [69] under a CC BY license, with permission from Dr. Italo Muntoni, original copyright 1983. Micrograph (PDC007) under normal transmitted light (top) showing no bacterial bioerosion but small areas of dark Wedl MFD surrounding micropores (bottom right and middle centre), and under polarised transmitted light (bottom) showing frequent, small bright (birefringent) white- and yellow-coloured inclusions in the micropores surrounding osteons.

Two samples presented full preservation of the bone microstructure (OHI = 5), with no evidence of bacterial attack: burial T9 at Titolo and burial T11 at Passo di Corvo (Fig 9). Both were primary burials. T9 was placed in a shallow pit grave with their trunk prone and legs flexed; some movement of the vertebrae and small bones of the hands and feet *in situ* is indicated as numerous bones were concreted when excavated. T11 was a prone deposition with their legs flexed at the knee either side of the trunk and placed in the bottom of a well [68]. Additional histotaphonomic features were observed in both samples. In T9, there were frequent ancient microcracks in the bone. In T11, there were occasional islands of Wedl MFD, observed on the margins and surrounding porous spaces in the bone, as well as pervading mineral concretions. When viewed under polarised transmitted light, both samples showed no collagen birefringence, indicating destruction of the organic bone phase. The loss of collagen is consistent with collagen hydrolysis through chemical degradation, a diagenetic pathway often encountered in burials in poor-draining or inundated soils [17, 27, 93].

## 5. Discussion

The objective of this study was to investigate the post-mortem biographies of individuals found in diverse contexts from Neolithic sites in Southeast Italy. This was carried out through histotaphonomic analysis of bone samples from articulated primary burials, semi-articulated primary or disturbed primary burials, and disarticulated bones, from 3 sites in Foggia and 1 site in Bari (Apulia). Decades of archaeological excavation and taphonomic studies have shown that the most archaeologically visible funerary rite was primary burial, and yet the most common fate of the dead was probably to end up dispersed as disarticulated fragments [1, 70, 94]. If this is the case, we ask what happened to the bodies of the dead before their remains were disturbed and transported across the landscape.

Bacterial bioerosion profiles varied considerably within each of the sites studied and the extent of bacterial attack was not related to the burial environment or feature. Samples from individuals deposited in similar burial features, or multiple samples from individuals in similar and different states of articulation deposited within the same feature repeatedly demonstrated different histotaphonomic characteristics. This supports the concept that early post-mortem treatment is at least partly responsible for the bacterial attack observed in these bones. Therefore, given the lack of evidence for a clear relationship between the burial environment and bacterial bioerosion, we explore the results of this study mostly through the lens of early post-mortem taphonomic histories, although we acknowledge that there are continuing uncertainties surrounding the aetiology of bacterial bioerosion.

### 5.1 Variable intra-site taphonomic histories

Importantly, this research has demonstrated evidence for variability in the extent of bacterial bioerosion between individuals and bone fragments deposited in the same features, or in similar areas and therefore under presumed similar geological and environmental conditions. This suggests that human remains at each site had variable associated taphonomic histories and the lack of significant inter-site differences in OHI scores also suggests these were similarly variable at each site. This variation is consistent with other archaeological studies [43, 44, 58] where it is assumed that bones of multiple individuals were accumulated or deposited after varied prior stages of mortuary processing. Given past associations between patterns of bacterial bioerosion and early taphonomy [14, 15, 28, 29], it is reasonable to explore possible interpretations which draw upon these associations. Under these models, the similarly variable patterns of bacterial bioerosion we observed across different sites would suggest that bodies at all sites were subject to a similar range of funerary treatments which produced comparable patterns of bacterial attack in the bones, although equifinality remains a substantial obstacle to interpretation.

Patterns of variable bacterial bioerosion observed across all sites were most similar to results obtained in other studies from remains deposited in fully or partially sheltered environments such as caves, tombs or covered pits [40, 41, 43, 54, 58]. These environments may have variably allowed or prevented access to skeletonising insects, exposing the bones to different levels of bacterial soft tissue decomposition. For example, although the Titolo burials were made in shallow graves, the pattern of anatomical connection observed on some skeletons during excavation and from elements which had become concreted together, suggests they could have decomposed in open spaces. The shallow graves may have contained minimal soil surrounding the body, and indeed many stones were present in the grave pits as well as atop the graves. At Masseria Candelaro, individuals T3 and P3/4A appear to have been placed in features which were open for an undefined period post-mortem (see below) [67]. Another individual who was placed in an open pit at Masseria Candelaro (P12 T2A, not included in this study), displayed an insect bore on their left humerus, suggesting that such features did provide access for invertebrates to colonise decomposing cadavers. The depositional environment in these cases appears to have produced a different set of circumstances for decomposition, which could have altered or slowed the effects of bacterial bioerosion. Some semi-articulated and articulated skeletons presented OHI scores of 2–3, which corresponds to the OHI scores assigned to most of the disarticulated bones in this study. Disarticulated bones, then, could have originated from contexts where bodies had been placed in open features or shallow graves immediately post-mortem, and their bones later retrieved and moved elsewhere.

Further support for this interpretation may be found in the identification of probable Wedl MFD, observed in 13 samples. Wedl MFD appear to be more common in archaeological human bones deposited in open environments such as caves or pits, suggesting that fungal spores may have been transported in the air [12, 41], although see [37] for a contrary perspective. Studies have shown that Wedl tunnels produced by fungal activity may not be present until at least one year post-mortem [38]. The observation of probable Wedl tunnels in a high number of samples in this study could indicate that some bodies were exposed in open features, perhaps for several years after death.

Therefore, the simplest explanation for the patterns of microbial bioerosion observed in the remains studied here is that the samples presenting extensive bacterial attack were buried intact in the ground initially, with some fragments eventually being removed from burials for secondary deposition, perhaps after a period of circulation. Other depositions, with an arrested profile of bacterial bioerosion, occasionally alongside Wedl MFD, were likely carried out in contexts such as caves or partially covered pits before some were later manipulated, disturbed and/or processed after they had partially or fully decomposed. However, problems of equifinality and the uncertainties about the timing of bacterial bioerosion and its relationship with soft tissue decomposition mean that other more complex scenarios could account for the variation we see.

### 5.2 Circulation and mobility

Given previous suggestions that the presence of cutmarks was related to locality at Grotta Scaloria [79], we wondered whether this relationship would be demonstrated at a wider scale across the region. Statistically, there was no association between bacterial bioerosion and whether an individual was local or not to their place of deposition at all sites except Titolo, where this could not be tested. This suggests that funerary practices were broadly shared across the region and whether an individual grew up, or even lived most of their life, around the area that they were buried in, did not influence the ways that their body or remains were processed after death. All sites included here (except Passo di Corvo, on current evidence [82]) contained individuals who were isotopically inferred to be local as well as non-local. This shows that changing residence patterns in adulthood, if not earlier, were common in Neolithic Southeast Italy. Frequent mobility probably enabled and strengthened shared cultural patterns over a wide area, such as we see reflected through the funerary practices. We interpret this mobility as a phenomenon which continued post-mortem, as indicated by the movement of bone fragments around sites, and the deposition of parts of bodies in different sites and features.

### 5.3 Primary burials with diverse taphonomic histories

Extensive bacterial bioerosion (low OHI scores) was expected for primary burials, given findings from previous studies [12, 40, 57]. However, we also expected to see differences in OHI scores between individuals in undisturbed primary burials and those displaying evidence of modifications which could indicate arrested putrefaction, such as hyperflexed limbs and cutmarks. Hyperflexion of the lower limbs may indicate tight wrapping of the body post-mortem, but can also be caused by delayed sediment infilling after decomposition [95]. The results of our analysis show that most articulated and semi-articulated primary burials underwent either arrested or extensive bacterial bioerosion, regardless of the extent of limb flexion or presence of cutmarks. Taphonomic evidence for extreme flexure of the lower limbs and/or early post-mortem defleshing did not affect the extent of bone bioerosion in these individuals. This is consistent with recent histotaphonomic analysis of remains from Çatalhöyük, where no distinct patterns were observed between delayed and non-delayed burials [44].

Several samples pertained to fragments or skeletons which presented cutmarks: GS21 (OHI = 4), PDC001 (T7, OHI = 2), PDC005 (T3, OHI = 1). At Passo di Corvo, some skeletons from primary burial contexts displayed cutmarks across numerous elements and yet were at least partly in anatomical connection when they were excavated [68, 70]. In the case of Passo di Corvo T3, the grave contained much of the skeleton in approximately its original position, but the skull and several other elements were absent; the removal of these bones may have occurred when the grave was reopened to cut and deflesh the decayed remains. T7 similarly contained an incomplete skeleton—the lower limbs were missing but some foot bones were present—although without detailed excavation records it is only possible to suggest that they represent either a disturbed primary burial or a secondary deposit of defleshed remains. Nevertheless, both burials demonstrate processing of the cadaver prior to full skeletonization, as well as the removal of selected bones. Similarly extensive but variable patterns of bacterial bioerosion have been observed in human remains from sheltered or open environments such as caves, tombs or pits [40, 43, 54, 58]. Some of the skeletons from Passo di Corvo may have been buried for a short time before being disturbed or may have been left to decompose in covered pits allowing for later access and manipulation. The factors responsible for the variability in bacterial bioerosion in these conditions are unclear but may be related to the specific environments produced by partial or sheltered burial.

For the disarticulated and cutmarked fragment from Grotta Scaloria, the minimal extent of bacterial bioerosion could indicate that the process of removing the element from the body occurred at an early stage post-mortem, when the individual has been buried for only a short time. An alternative possibility is that the body had been initially placed in an open or partially sheltered context that still allowed for some removal of soft tissue by skeletonising invertebrates before the body was fully artificially defleshed. In contrast, at Passo di Corvo, defleshing may have been a later part of the ritual practice, or else the bodies could have been placed in sheltered contexts. Given that the skeletons displaying cutmarks appear to have been placed in correct anatomical position, it seems likely that they were defleshed while *in situ* in the grave.

Two primary burials demonstrated full histological preservation which we argue was likely caused by collagen hydrolysis: Passo di Corvo T11 and Titolo T9. The burial of Passo di Corvo T11 in a well suggests that, while the well was presumably disused at the time of deposition, the feature was deep and not free-draining, and may have continued to be inundated. Titolo T9, on the other hand, was placed in a shallow pit and, although the soil cover was probably well-draining, the feature may have been prone to waterlogging. Comparison may be found with a similar case of presumed collagen hydrolysis from a Medieval burial in Apigliano, also located in Apulia [96]. The authors inferred that frequent cycles of wetting, drying, and periodic high summer temperatures may have caused collagen hydrolysis. However, it is curious that T9 should be the only burial analysed histologically from Titolo (5/12 individuals studied) displaying chemical hydrolysis, as the burial feature is consistent with the other graves from the site. T9 is the earliest dated burial (5/12 individuals dated) from Titolo; the median date (5706 cal BC, 95.4% probability) is 208 years earlier than the median date for the next-earliest dated burial (T2, 5498 cal BC, 95.4% probability) [62]. It is possible to speculate that this body could have been treated and/or deposited elsewhere initially and moved to the grave at Titolo in a state of semi-articulation, perhaps representing a founding ancestor for the necropolis. Unfortunately, given the poor preservation of the skeletal remains, it is not possible to evaluate this further through archaeothanatological observations of the position of the skeleton and joint articulations *in situ*.

## 6. Conclusion

Histotaphonomic analysis of bone microstructure is an important tool for funerary taphonomic research. Macroscopic taphonomic analysis provides insights into long-term patterns of post-mortem and post-depositional treatment, based upon the accumulation of centuries or millennia of taphonomic alterations from diverse agents. Histotaphonomy potentially facilitates finer scale analyses of bone diagenesis in the short-term post-mortem. This may relate to the scale of decades rather than years [22], and current research is divided as to the relationship between decomposition, depositional environments and bone bioerosion [29, 35–37]. Further actualistic research is warranted to better understand the timing at which bacterial bioerosion begins post-mortem, the rate and duration over which it accumulates, and the origins of Wedl MFD in human bones placed in different climates, environments, and depositional circumstances. The combination of both macroscopic and microstructural taphonomic analyses thus provides the opportunity to study deathways as integrated ritual programmes and multi-stage processes.

Within the programme of Neolithic deathways in Southeast Italy, primary depositions have frequently been prioritised in bioarchaeological analyses and archaeological syntheses, which have often considered them to be straightforward ritual contexts. More recent taphonomic analyses have shown that there was variation even within the category of primary burials [70, 97]. Further questions have arisen from the evidence for the retrieval and circulation of bone, and secondary deposition of disarticulated bones [64]. Specifically, it is important to establish whether these interlinked processes mark different possible stages of the funerary ritual, or whether the deposition of disarticulated remains represents the end-stage of distinct processes such as excarnation.

Here, we have attempted to address the relationship between these diverse funerary circumstances, to form an understanding of deathways in their regional setting. Histotaphonomic analyses of remains from a variety of depositional contexts and states of articulation supports previous research which has emphasised the diversity of funerary practices within sites [1, 70], and sheds light on the potential prior treatment of individuals whose remains became disarticulated. Our analysis shows signatures of arrested and extensive bacterial bioerosion in bones from articulated, semi-articulated and disarticulated states (Fig 5). These results contradict some studies which have suggested a straightforward relationship between the extent of bacterial attack according to whether remains are articulated or disarticulated [14, 28, 54]. Articulated/partially articulated and disarticulated remains in Iron Age Britain showed clear differences in histological preservation, suggesting distinct post-mortem treatment of remains which ended up in different contexts. Conversely, similar variation in histological characteristics for articulated, semi-articulated and disarticulated bones in Neolithic Southeast Italy suggests that diverse final modes of deposition may be linked by the retrieval of bones from exposed or buried individuals. Variation in histological preservation is probably attributable to circumstances of partially sheltered burial and variable exposure to invertebrates during early decomposition. Cycles of open-air exposure and burial in aerobic soils, for example in primary burials from which bones were removed post-skeletonisation, or deposition in open pits which progressively infilled, could be responsible for arrested bacterial bioerosion and Wedl destruction.

Environmental conditions between the cave and village sites also resulted in some distinct histological features. At Grotta Scaloria, concretions on and within bone fragments were much more prevalent than elsewhere. In contrast, probable iron oxide staining was more frequent in samples from the village sites than at Grotta Scaloria, where clayey soils predominated. Nevertheless, the presence of some fragments with staining at Grotta Scaloria does suggest earlier histories of deposition in different soils. We also identified evidence for collagen hydrolysis in two individuals, probably associated with specific burial features which were prone to waterlogging.

By integrating OHI scores with Strontium isotopic measurements as a proxy for locality to a subset of these data, we showed that there was no clear relationship between whether an individual was isotopically local to their place of deposition and their mode of funerary treatment. This line of analysis is worth pursuing in future studies of funerary practices, including histotaphonomic research. For example, Haddow et al. [44] speculate as to whether increased mobility of a subset of the adult population, or during a particular time period, is responsible for the variation observed in histotaphonomic characteristics at Çatalhöyük.

In combination, these results contribute to the ongoing discussion about the complexities of interpreting bacterial bioerosion and other histotaphonomic features [35, 36, 44]. We provide suggestions for the particularities of our data through close attention to archaeological context. These suggestions may be testable in future actualistic research, although such studies may need to be carried out over longer timescales than is usually common. By attending to the contextual evidence for diverse conditions of deposition alongside histotaphonomic features—including sheltered or closed contexts, open air exposure, temporarily open burial features and secondary deposition—we demonstrate possible pathways for the retrieval and mobility of disarticulated bone fragments. This was a widespread practice beyond southeastern Italy during the 6^th^–5^th^ millennia BCE and is evident across much of later European prehistory. Alongside others [43, 54, 59, 98], we argue that this practice deserves further attention, and histotaphonomic analysis of disarticulated remains in their regional context provides one method for doing so.

## Supporting information

S1 File Available Strontium isotopic measurements. (XLSX)

S2 File Thin section micrographs. (PDF)
