# Supplementary material for: Histotaphonomic analysis of bone bioerosion reveals a regional framework of diverse deathways in the Neolithic of Southeast Italy
Source: PLoS One. 2024 Jun 6;19(6):e0304058. doi: 10.1371/journal.pone.0304058 (PMC11156350; doi:10.1371/journal.pone.0304058)
Supplement: S2 File — (PDF) [file pone.0304058.s002.pdf]

Normal transmitted light, OHI score given	Polarised transmitted light, BI score given
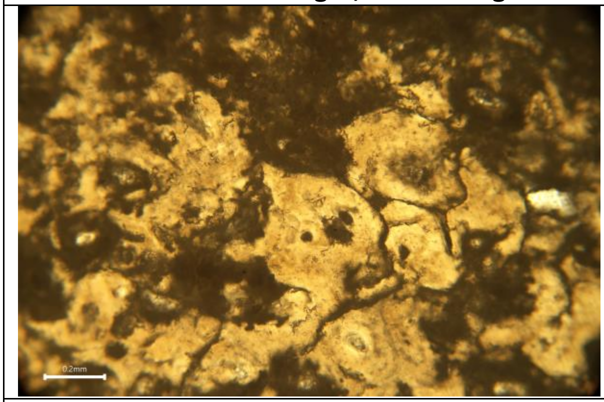	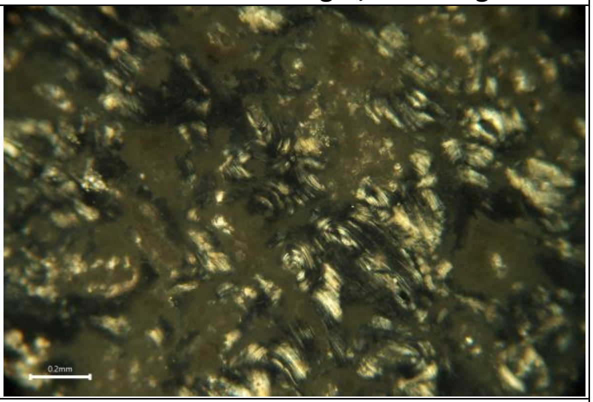
Figure 1: GS2, OHI: 2, GHI: 1	Figure 2: GS2, BI: 0.5
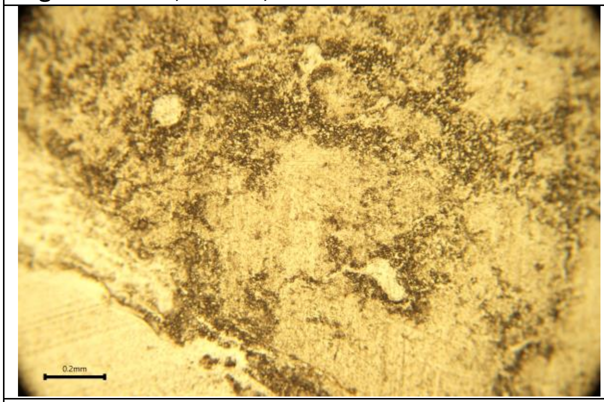	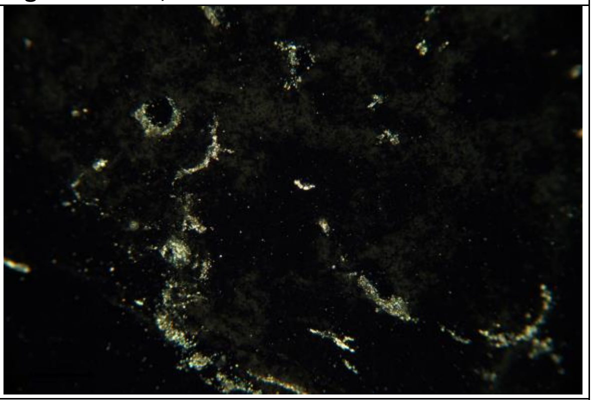
Figure 3: GS5, OHI: 1, GHI: Not scored	Figure 4: GS5, BI: 0
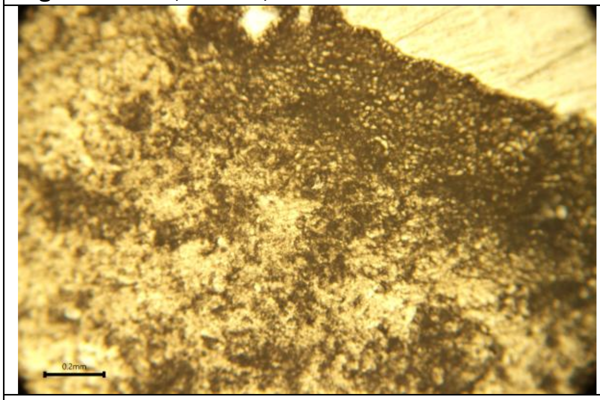	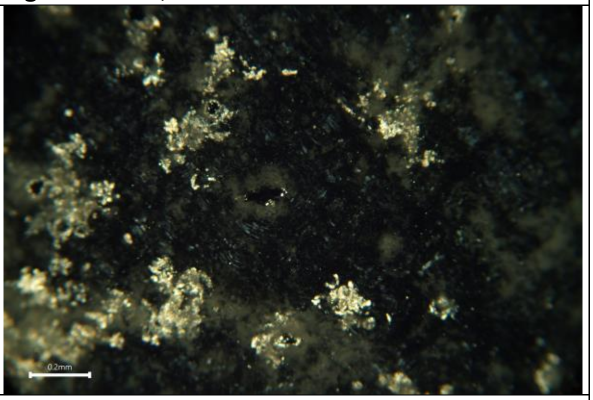
Figure 5: GS6, OHI: 1, GHI: 1	Figure 6: GS6, BI: 0
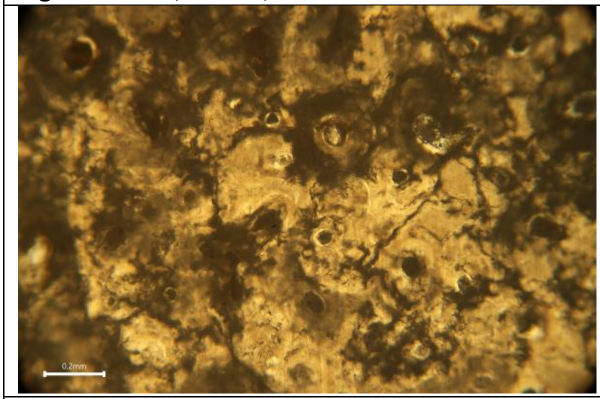	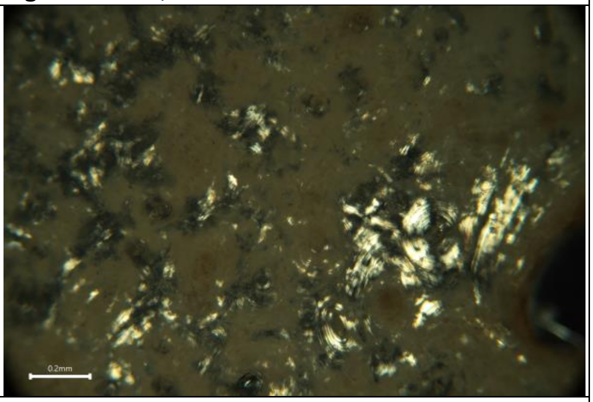
Figure 7: GS9, OHI: 1, GHI: 1	Figure 8: GS9, BI: 0

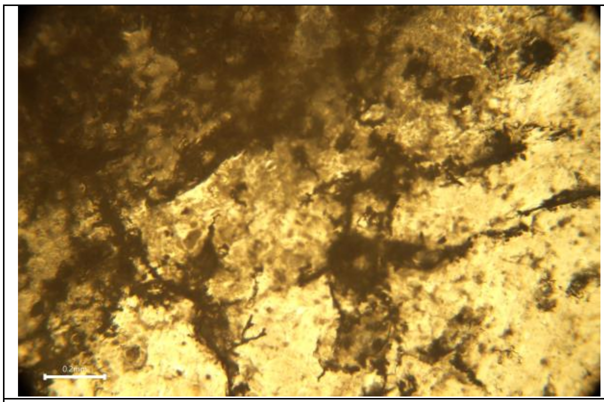

Figure 9: GS13, OHI: 3, GHI: 2

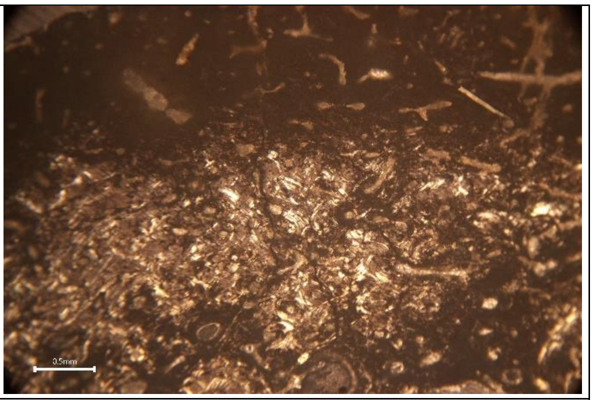

Figure 10: GS13, BI: 0.5

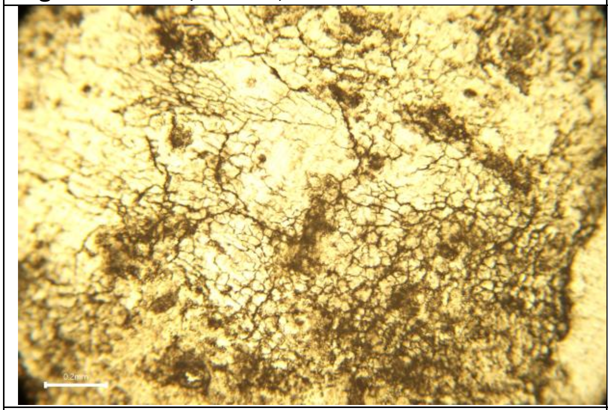

Figure 11: GS14, OHI: 3, GHI: 2

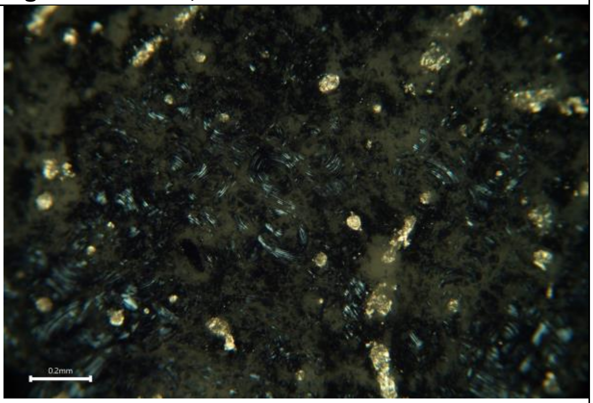

Figure 12: GS14: BI: 0.5

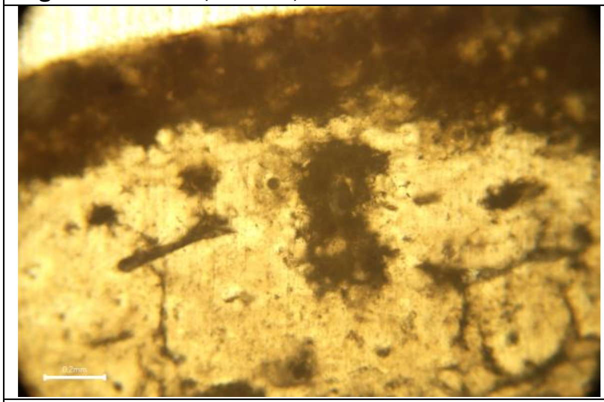

Figure 13: GS16, OHI: 3, GHI: 2

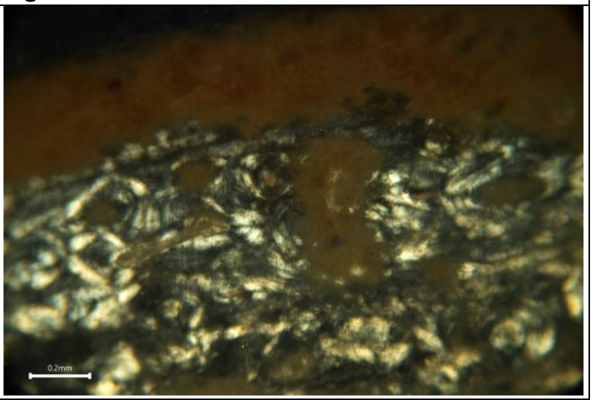

Figure 14: GS16, BI: 0.5

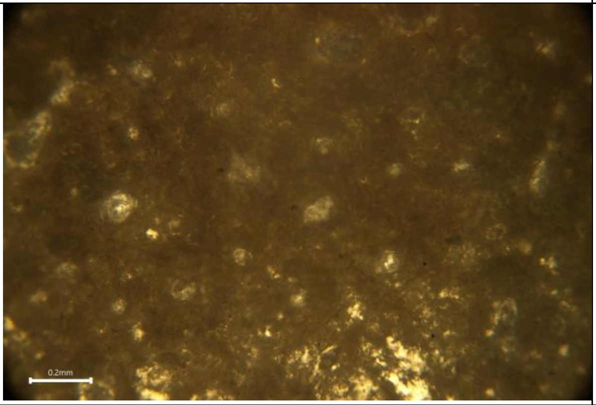

Figure 15: GS19, OHI: 2, GHI: 1

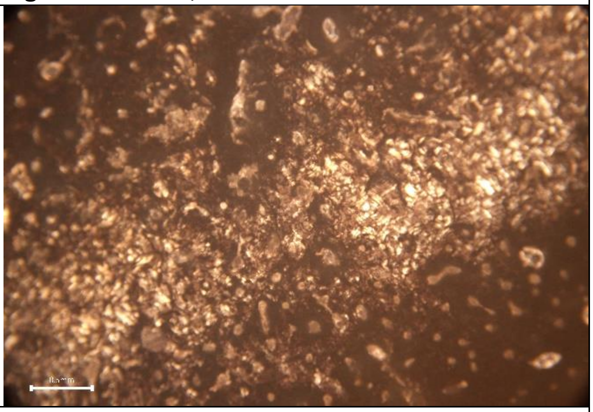

Figure 16: GS19, BI: 0.5

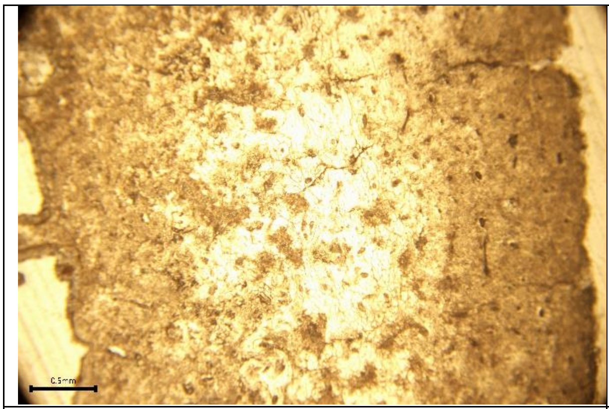

Figure 17: GS20, OHI: 2, GHI: 1

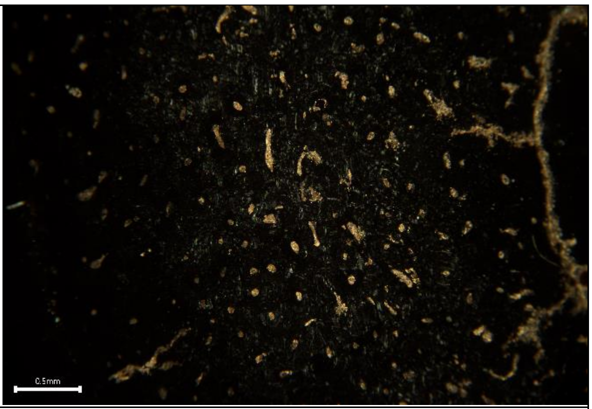

Figure 18: GS20, BI: 0.5

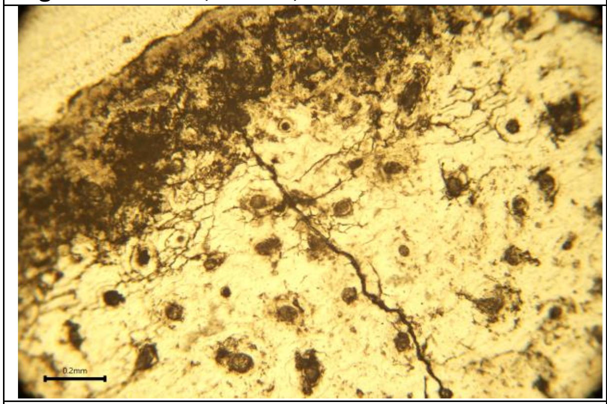

Figure 19: GS21, OHI: 4, GHI: 3

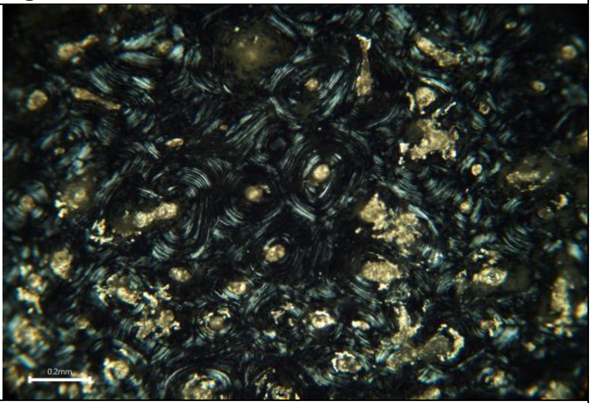

Figure 20: GS21, BI: 0.5

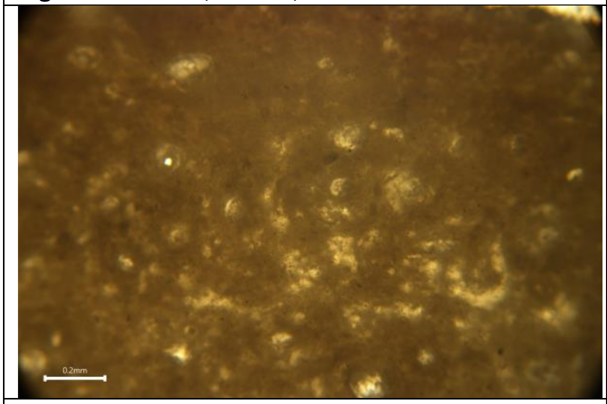

Figure 21: GS22, OHI: 2, GHI: 1

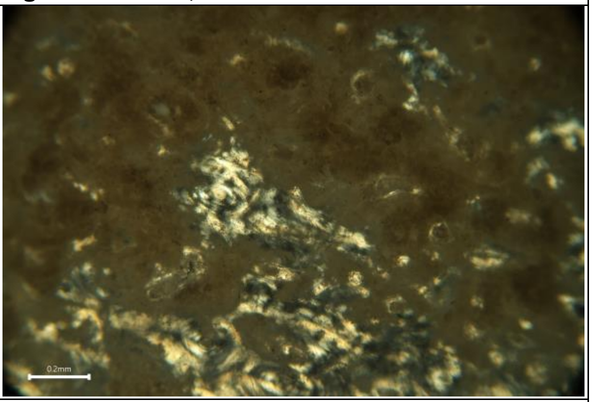

Figure 22: GS22, BI: 0.5

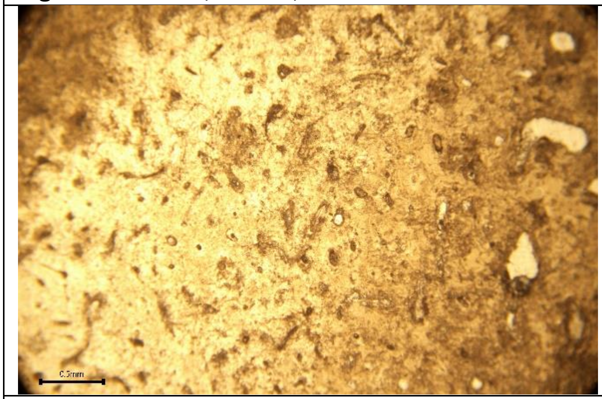

Figure 23: GS25, OHI: 2, GHI: 1

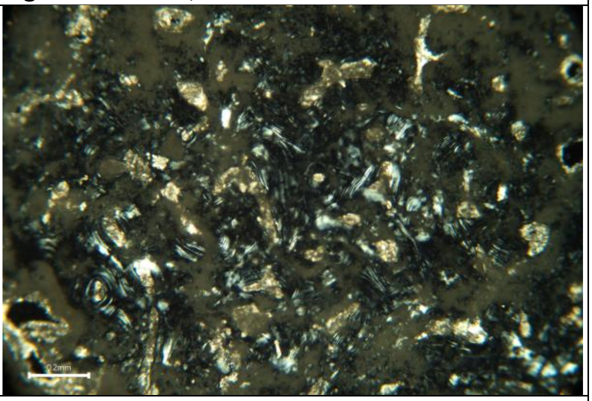

Figure 24: GS25, BI: 0.5

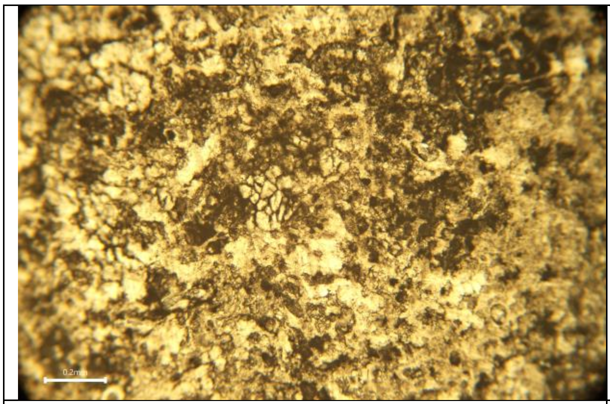

Figure 25: GS26, OHI: 2, GHI: 2

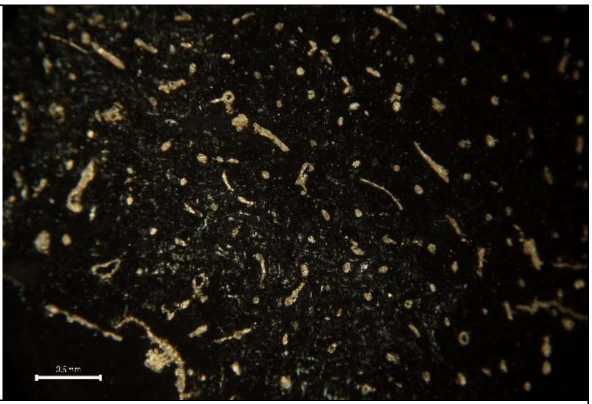

Figure 26: GS26, BI: 0.5

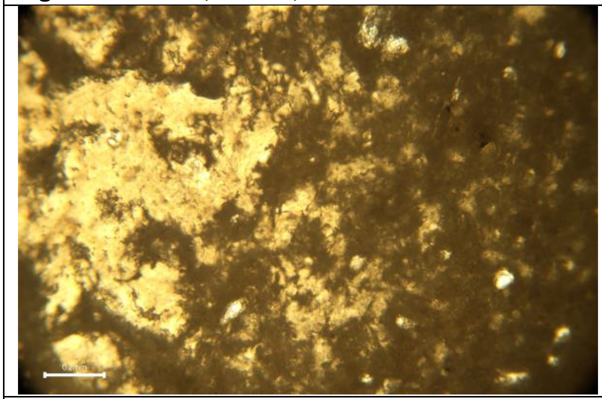

Figure 27: GS28, OHI: 2, GHI: 2

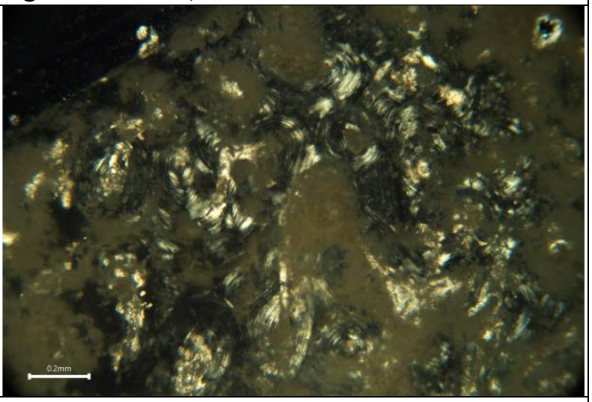

Figure 28: GS28, BI: 0.5

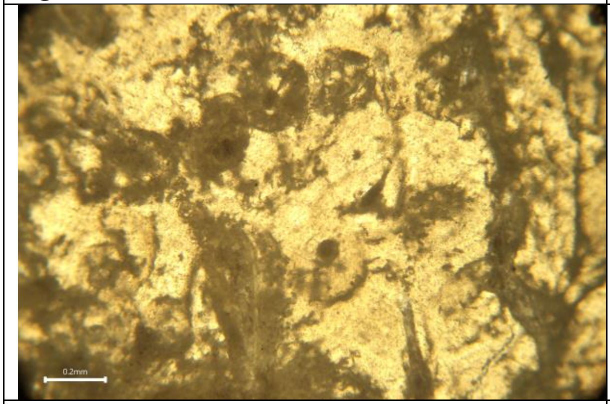

Figure 29: GS31, OHI: 2, GHI: 1

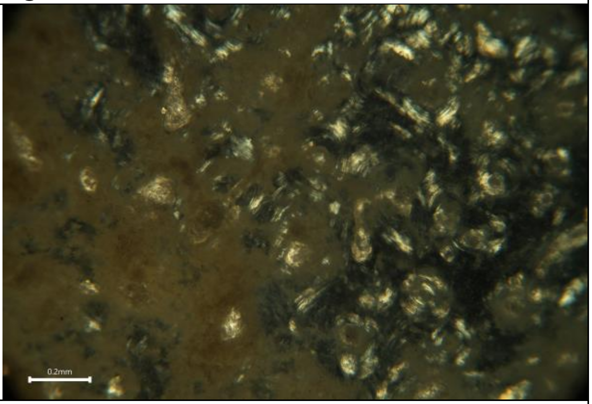

Figure 30: GS31, BI: 0.5

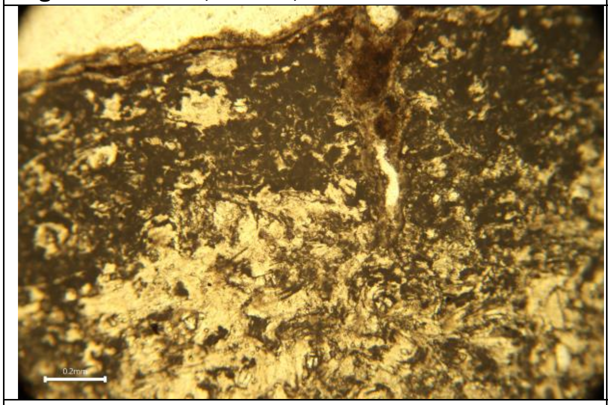

Figure 31: GS36, OHI: 0, GHI: 1

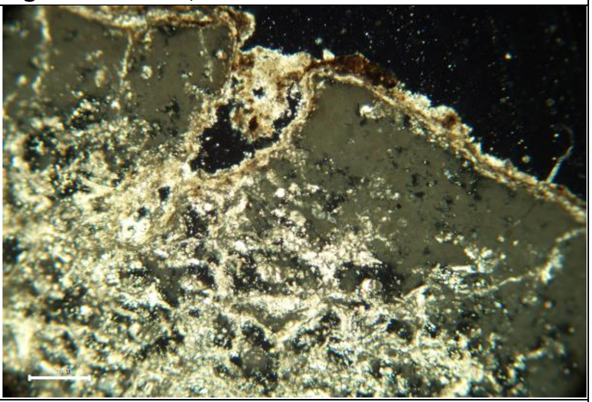

Figure 32: GS36, BI: 0

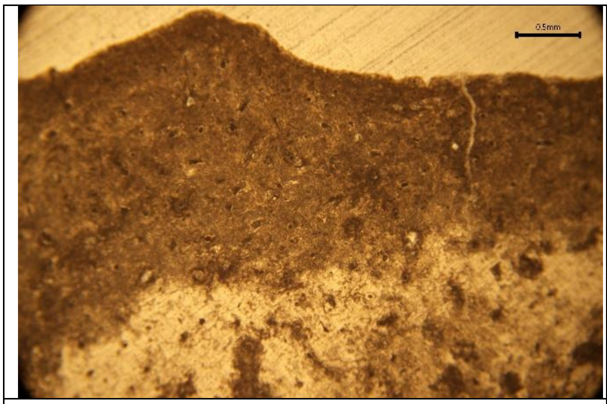

Figure 33: GS40, OHI: 2, GHI: 2

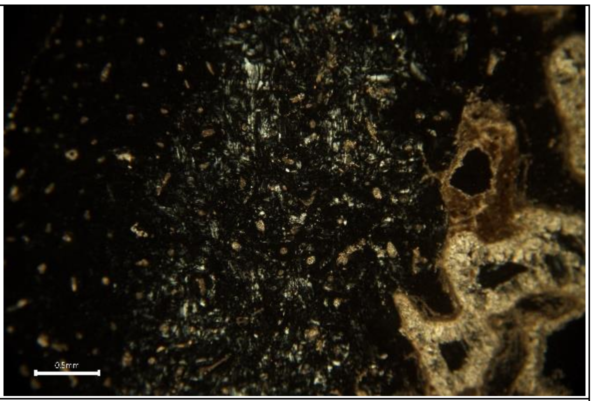

Figure 34: GS40, BI: 0.5

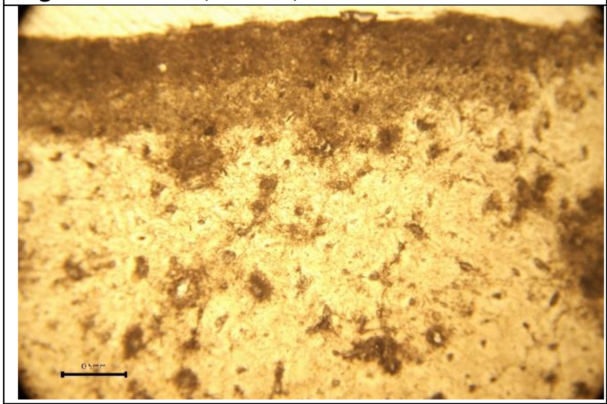

Figure 35: GS41, OHI: 3, GHI: 3

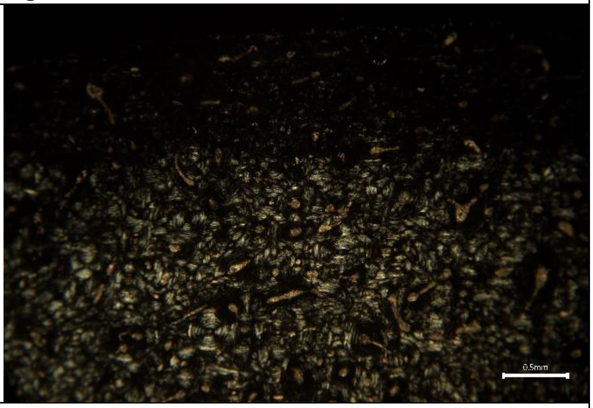

Figure 36: GS41, BI: 0.5

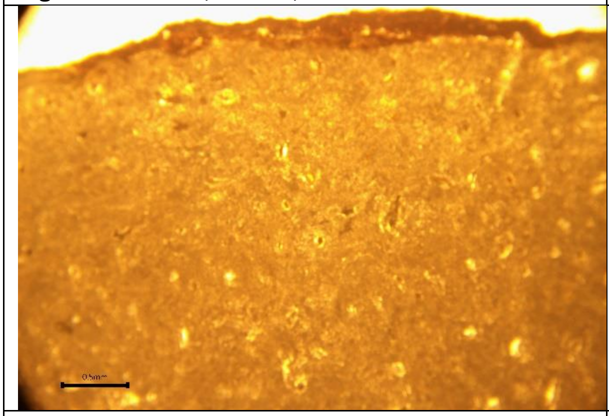

Figure 37: PAL011, OHI: 0, GHI: 0

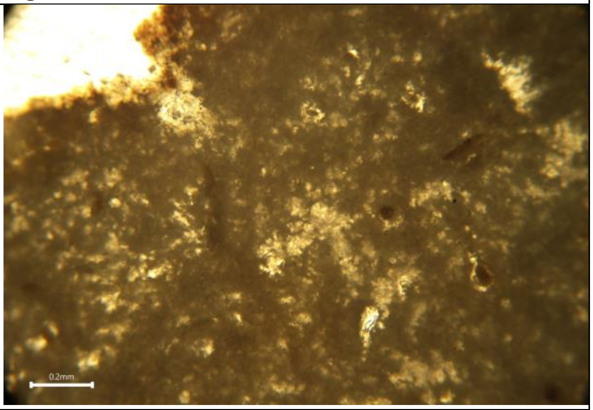

Figure 38: PAL011, BI: 0

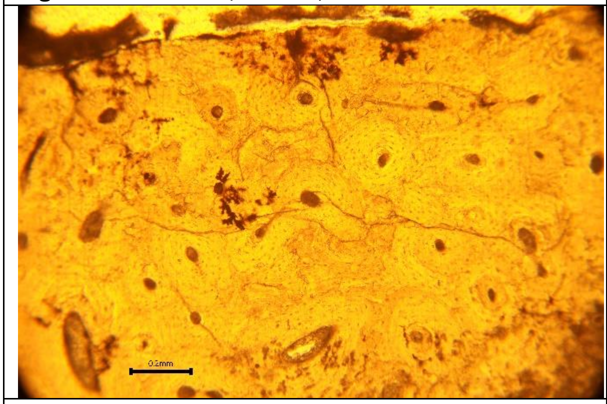

Figure 39: PAL012, OHI: 2, GHI: 2

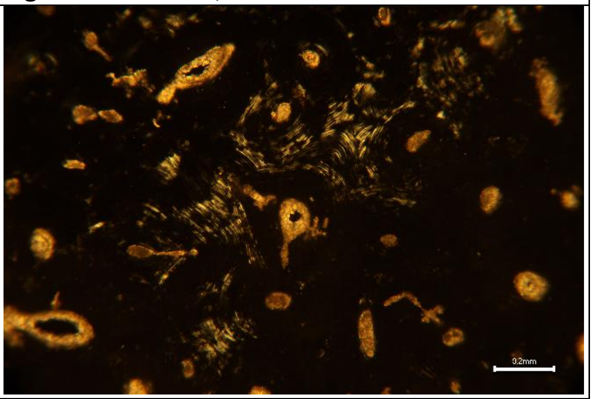

Figure 40: PAL012, BI: 0.5

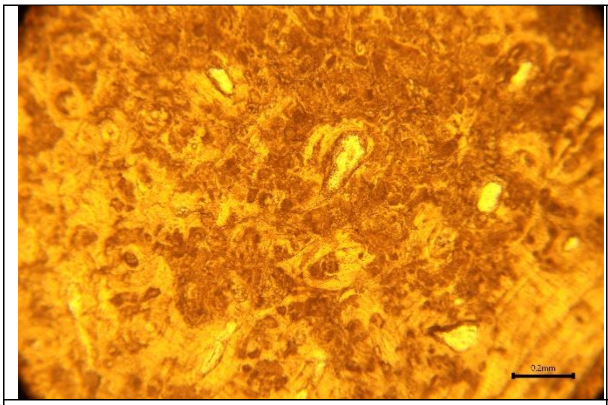
Figure 41: PAL013, OHI: 2, GHI: 1

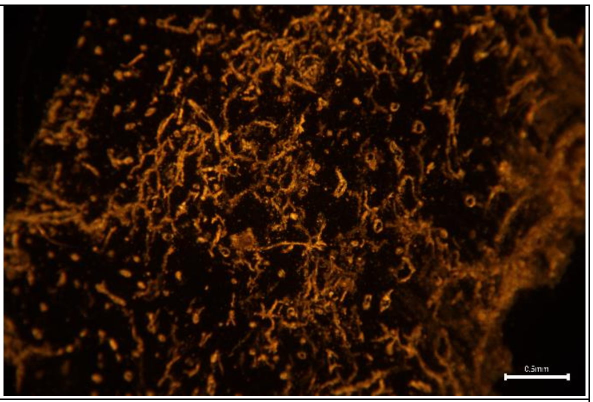
Figure 42: PAL013, BI: 0

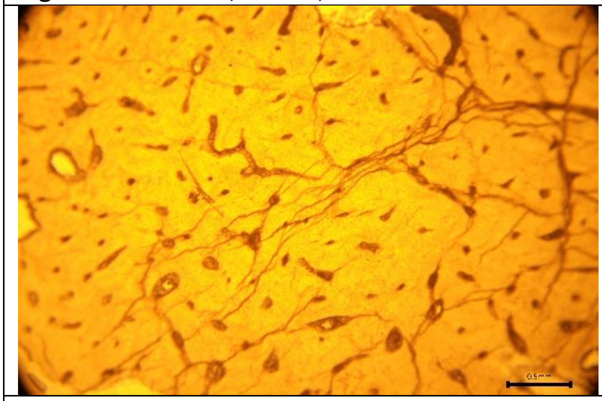
Figure 43: PAL014, OHI: 5, GHI: 4

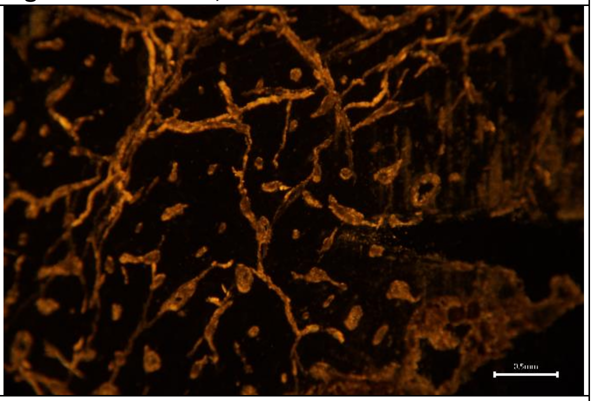
Figure 44: PAL014, BI: 0

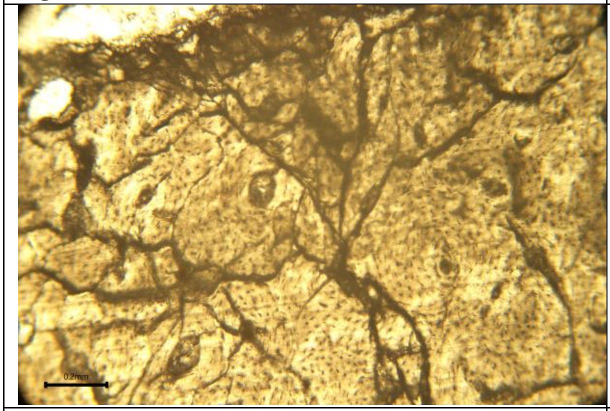
Figure 45: PAL015, OHI: 1, GHI: 1

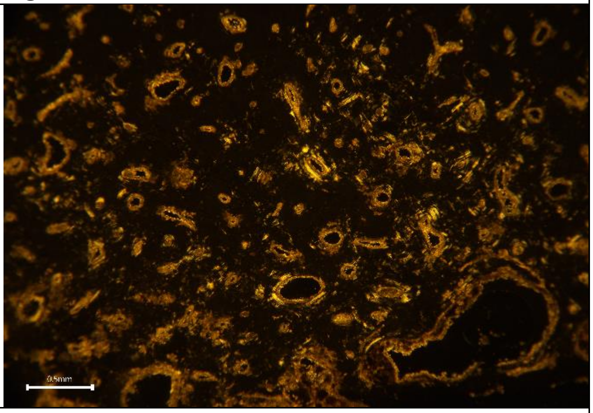
Figure 46: PAL015, BI: 0.5

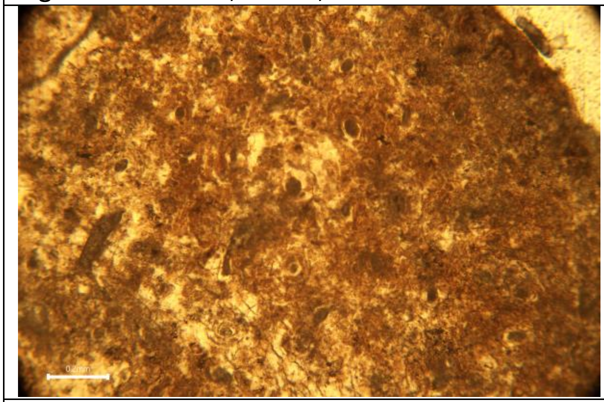
Figure 47: CND019, OHI: 3, GHI: 2

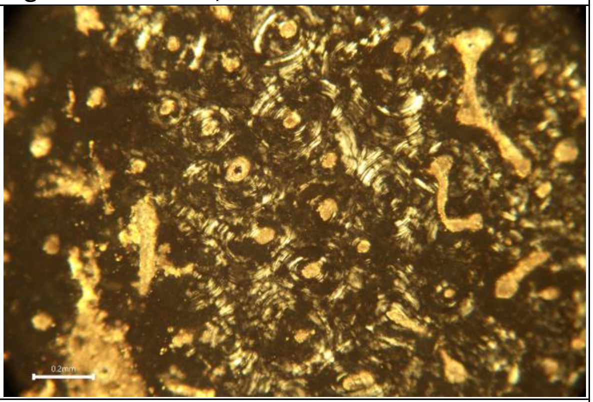
Figure 48: CND019, BI: 0.5

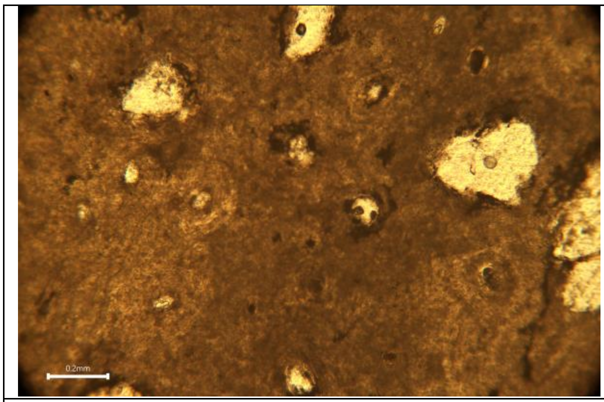

Figure 49: CND020, OHI: 1, GHI: 1

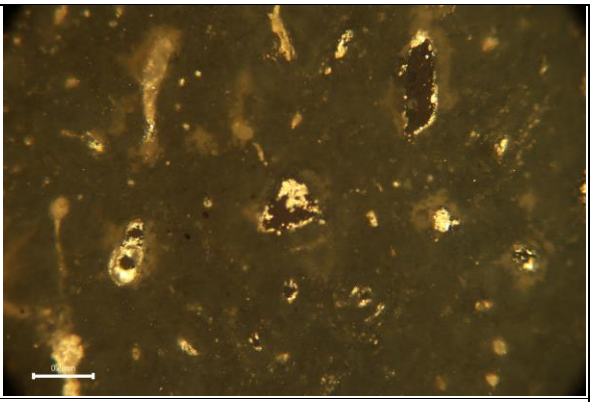

Figure 50: CND020, BI: 0

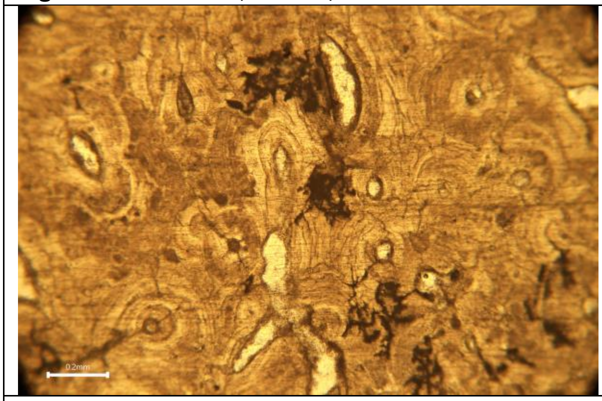

Figure 51: CND021, OHI: 4, GHI: 3

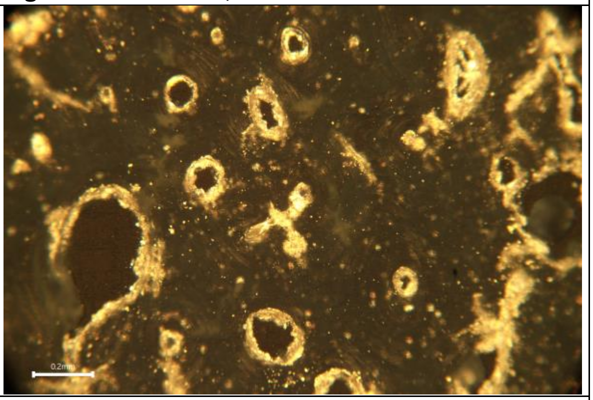

Figure 52: CND021, BI: 0

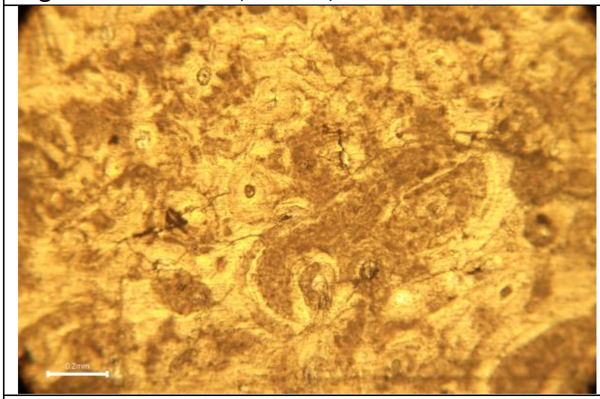

Figure 53: CND022, OHI: 3, GHI: 2

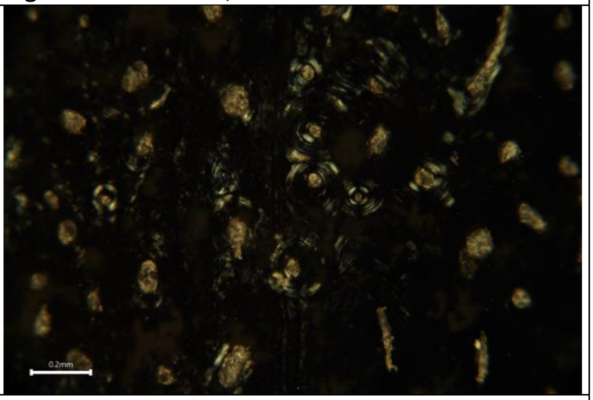

Figure 54: CND022, BI: 0.5

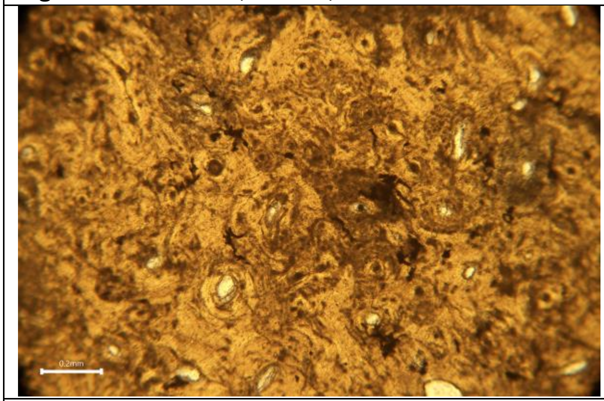

Figure 55: CND023, OHI: 3, GHI: 2

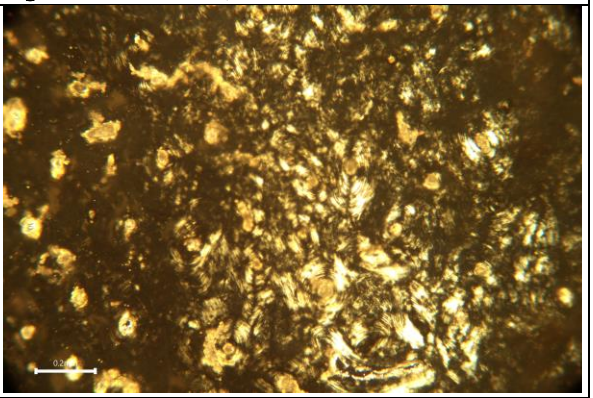

Figure 56: CND023, BI: 0.5

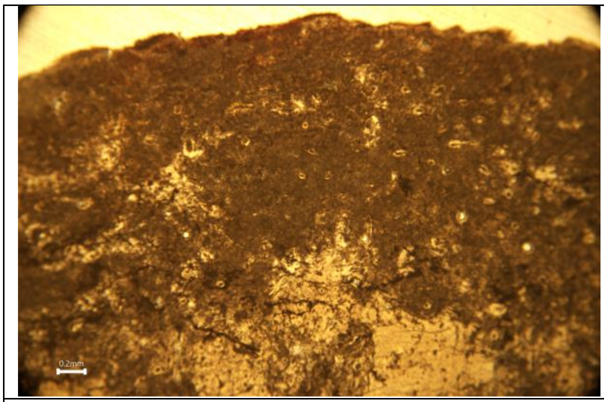

Figure 57: CND024, OHI: 2, GHI: 2

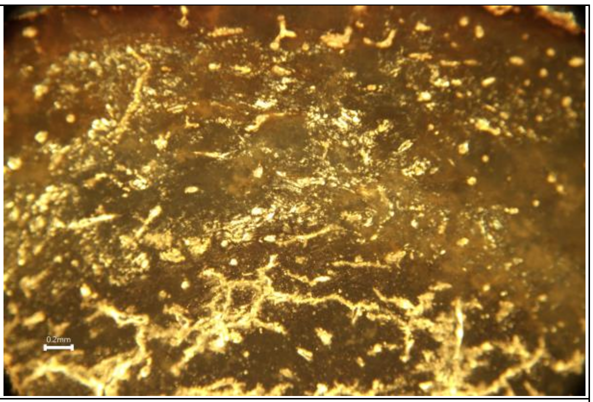

Figure 58: CND024, BI: 0.5

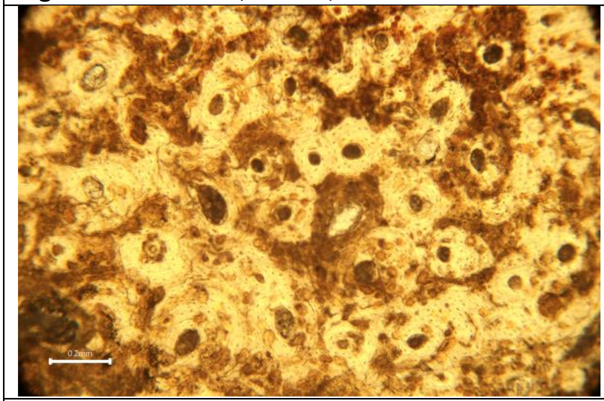

Figure 59: CND025, OHI: 2, GHI: 2

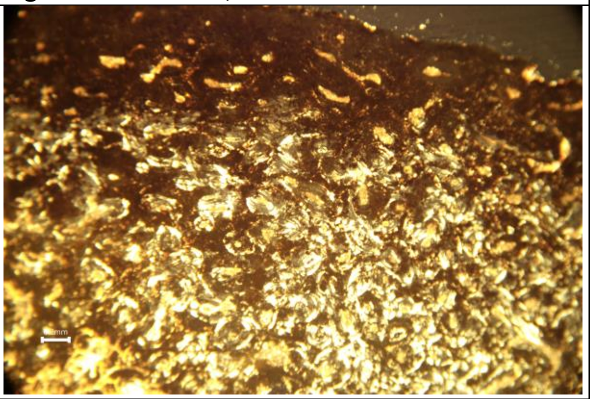

Figure 60: CND025, BI: 0.5

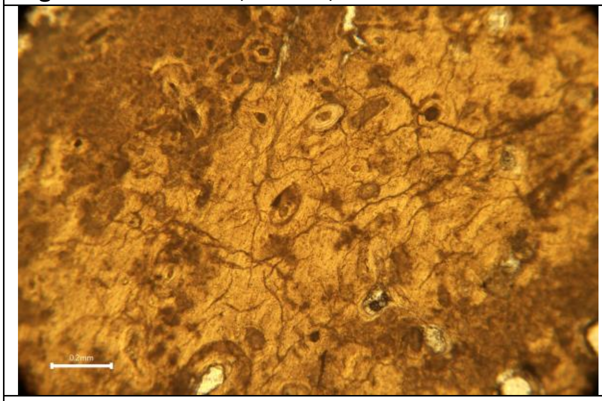

Figure 61: CND026, OHI: 1, GHI: 1

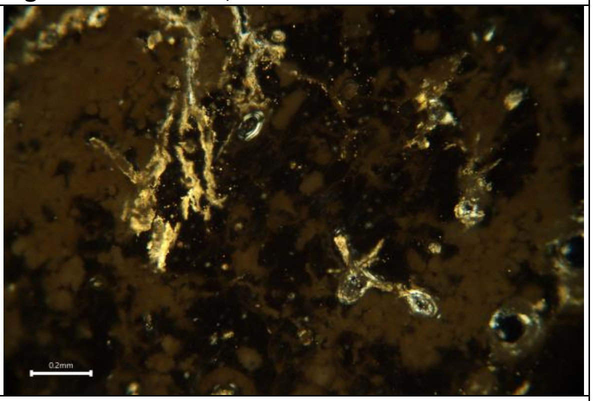

Figure 62: CND026, BI: 0.5

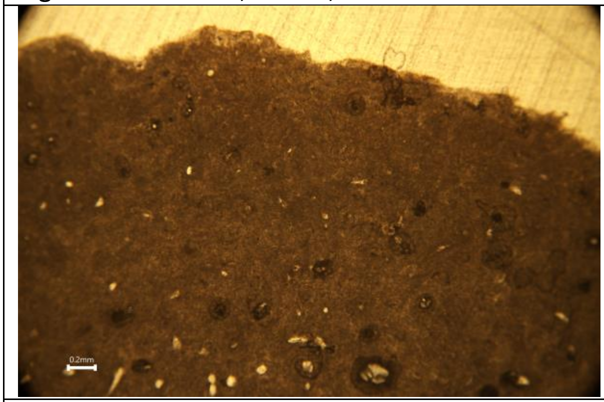

Figure 63: CND027, OHI: 0, GHI: 0

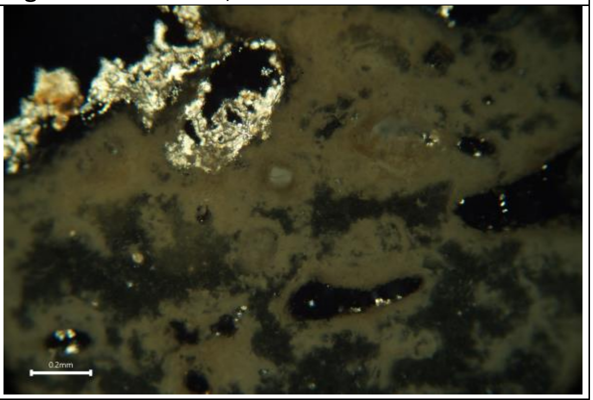

Figure 64: CND027, BI: 0

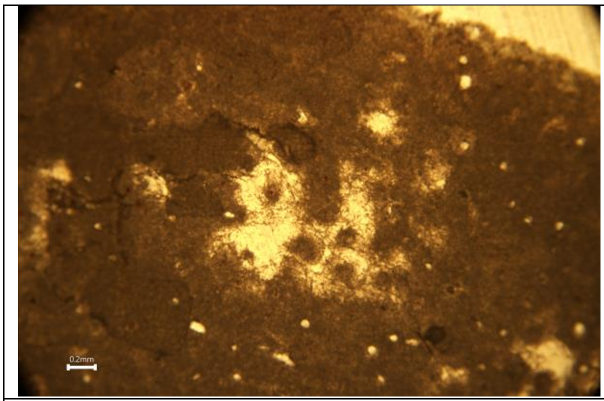

Figure 65: CND028, OHI: 1, GHI: 1

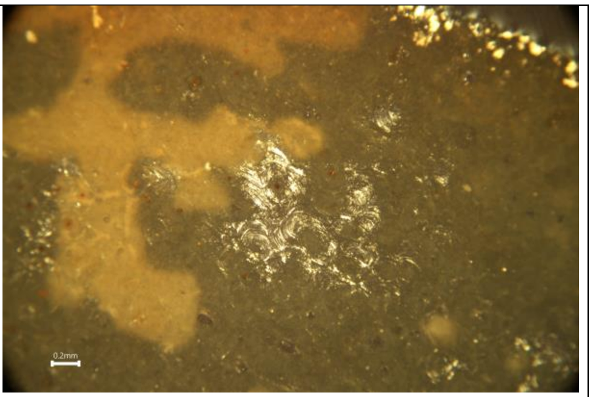

Figure 66: CND028, BI: 0.5

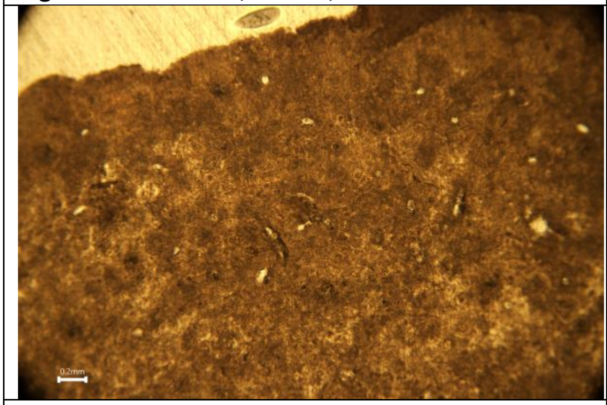

Figure 67: CND029, OHI: 2, GHI: 2

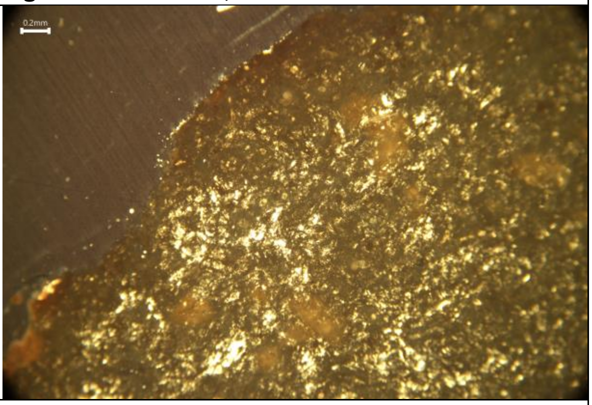

Figure 68: CND029, BI: 0.5

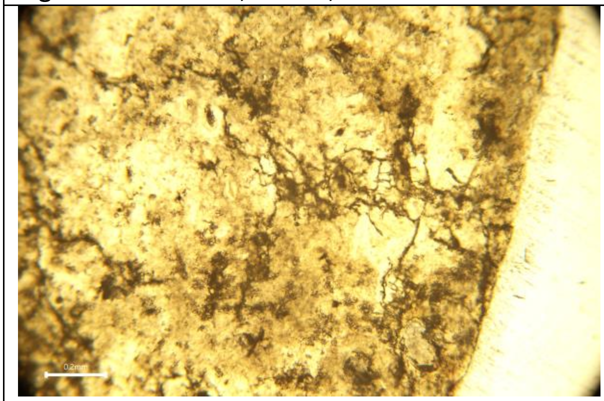

Figure 69: PDC001, OHI: 2, GHI: 1

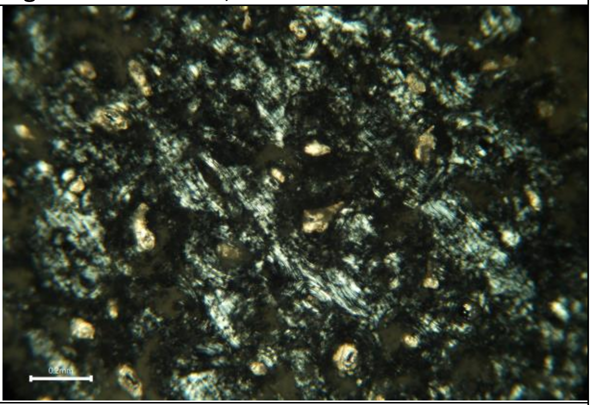

Figure 70: PDC001, BI: 0.5

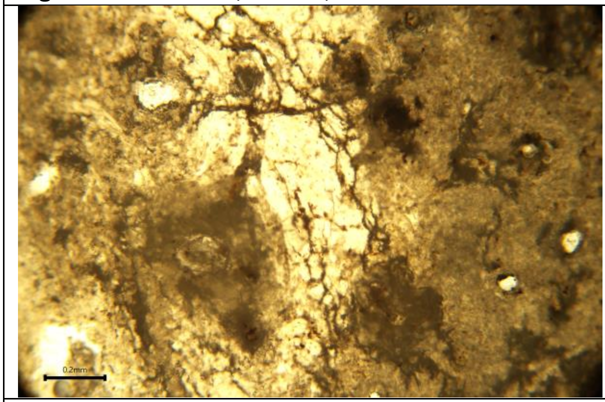

Figure 71: PDC002, OHI: 1, GHI: 1

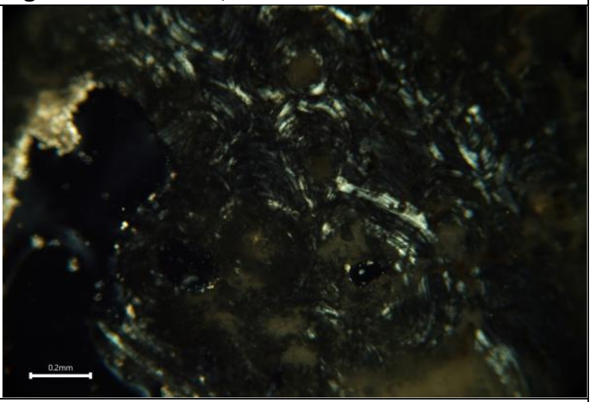

Figure 72: PDC002, BI: 0

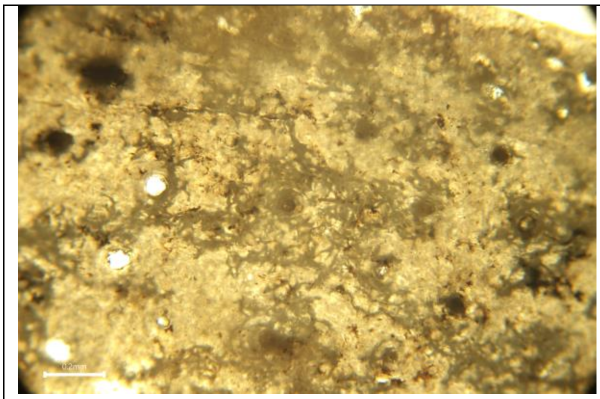

Figure 73: PDC004, OHI: 1, 1

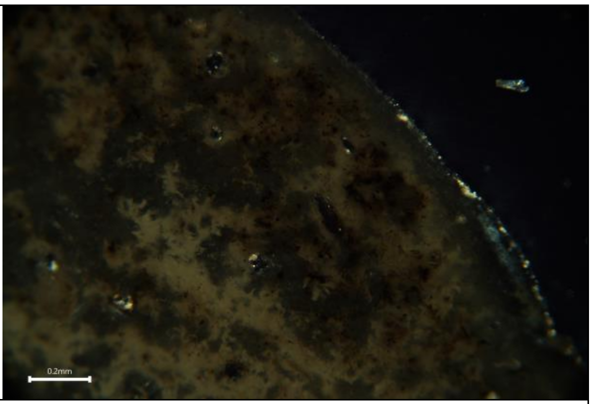

Figure 74: PDC004, BI: 0

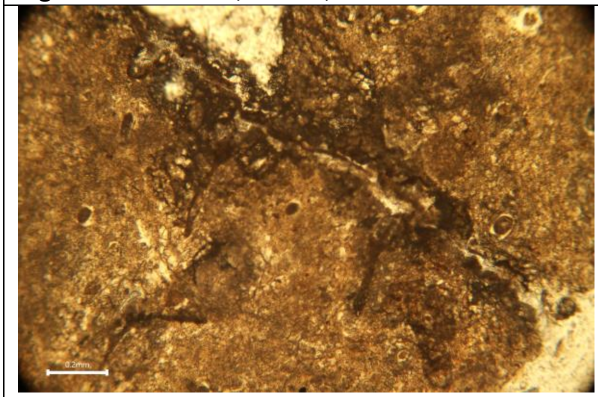

Figure 75: PDC005, OHI: 1, GHI: 1

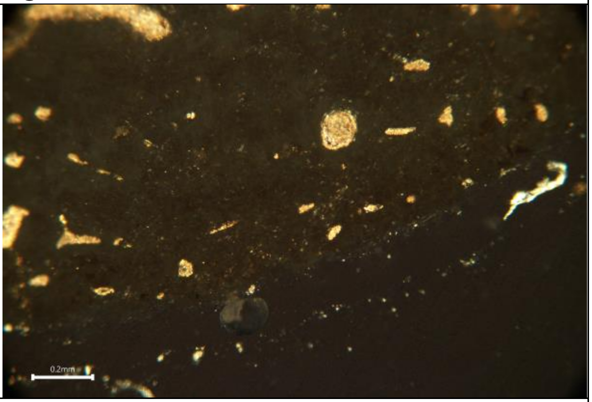

Figure 76: PDC005, BI: 0.5

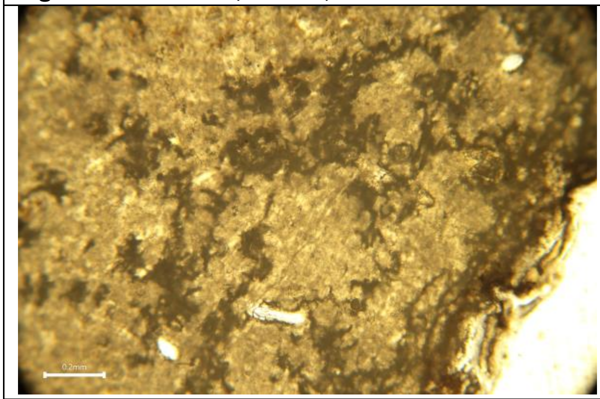

Figure 77: PDC006, OHI: 0, GHI: 0

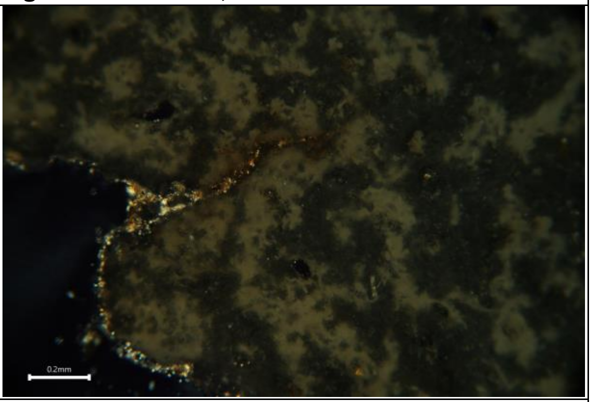

Figure 78: PDC006, BI: 0

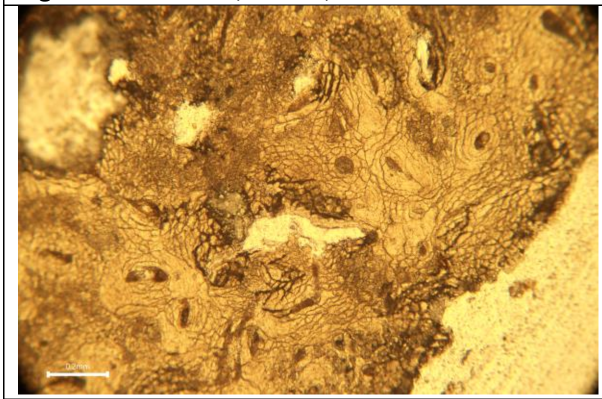

Figure 79: PDC007, OHI: 5, GHI: 4

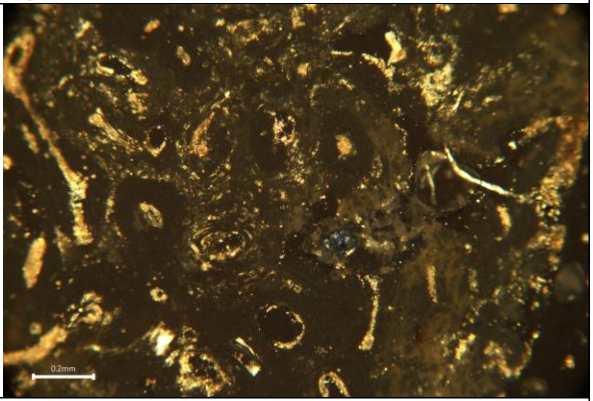

Figure 80: PDC007, BI: 0
